# Computer-Aided Drug Design for Immunosuppressive Tumor Microenvironment Reprogramming: A Critical Review

**DOI:** 10.3390/ijms27219766

**Published:** 2026-10-30

**Authors:** Jihoon Moon, Jehyeok Rew, Hyun Kil Shin, Kyoung Song, Hyosun Cho, Joohee Jung, Minjung Kyung

**Affiliations:** 1 Department of Data Science, Duksung Women’s University, Seoul 01369, Republic of Korea; jmoon25@duksung.ac.kr (J.M.); jhrew@duksung.ac.kr (J.R.); 2 Department of AI-Based Drug Development, Duksung Women’s University, Seoul 01369, Republic of Korea; phdgil@duksung.ac.kr; 3 College of Pharmacy, Duksung Women’s University, Seoul 01369, Republic of Korea; songseoul17@duksung.ac.kr (K.S.); hyosun1102@duksung.ac.kr (H.C.); 4 Duksung Innovative Drug Center, Duksung Women’s University, Seoul 01369, Republic of Korea; 5 Department of Statistics, Duksung Women’s University, Seoul 01369, Republic of Korea

**Keywords:** tumor microenvironment, computer-aided drug design, molecular docking, molecular dynamics, structure-based drug design, virtual screening, multi-omics, artificial intelligence, combination therapy, precision oncology

## Abstract

How can cellular interactions in solid tumors guide molecular interventions against immune suppression? This critical review examines how computer-aided drug design (CADD) connects immunosuppressive tumor microenvironment (TME) states to testable molecular hypotheses. We distinguish observational artificial intelligence (AI) predictions from molecular intervention hypotheses and examine the experimental evidence required to evaluate them. We reviewed patient-derived models, spatial and multi-omics profiling, molecular design, simulation, and pharmacology using literature identified through 15 October 2026. Adenosine-receptor antagonist discovery and generative protein-binder design illustrated how computational methods contributed to experimentally tested molecular candidates. Native-state structural studies, stromal delivery, and protein degradation broadened the approaches considered. We organized these findings into a proposed framework encompassing in vivo and patient-derived systems, multi-omics mapping, phenotyping of TME states, actionable AI integration, combination-therapy prioritization, and translational testing (IMPACT). A retrospective analysis of a phase III trial illustrated how the framework connects clinical outcomes to questions about mechanism, patient selection, and exposure. The synthesis clarifies the evidence required to connect molecular binding, target engagement, and exposure to patient benefit. IMPACT provides a structure for relating biological context to molecular design and specifying hypotheses for prospective evaluation.

## 1. Introduction

Precision oncology increasingly links molecular profiles with spatial, imaging, structural, and clinical information [1,2,3,4,5]. Patient-derived studies provided opportunities to test treatment responses in experimentally accessible tumor material [6,7,8,9]. Spatial and single-cell studies identified multicellular neighborhoods, fibroblast programs, and immune-cell states associated with clinically relevant tumor microenvironment (TME) phenotypes [10,11,12,13]. These findings raise a practical question: which features of an observed tumor state can guide a molecular intervention, and what evidence is needed to test that intervention? Answering this question requires a distinction between biological relevance and molecular targetability. An association with immune suppression can nominate a target without establishing whether a drug can reach or modulate it. Molecular targetability depends on the target’s structure, cellular location, available therapeutic modality, and achievable exposure. A useful design hypothesis connects these conditions to a defined mechanism that can be tested experimentally.

Computer-aided drug design (CADD) helps formulate this molecular hypothesis. Experimentally determined and predicted structures provide information about target geometry, with uncertainty around local pockets, interfaces, and conformational states [14,15]. Docking and virtual screening generate candidate poses and prioritize compounds within the limits of their sampling and scoring methods [16,17,18,19]. Deep-learning studies extended pose generation, while benchmark analyses identified physical invalidity, dataset overlap, and limited generalization as unresolved problems [20,21,22,23]. Molecular dynamics (MD) and enhanced sampling examine conformations that static structures can miss [24,25,26]. Free-energy calculations estimate relative molecular energetics when sampling and transformation design are adequate [27,28,29,30]. Quantum mechanics/molecular mechanics (QM/MM) treats electronic changes that classical models cannot represent explicitly [31,32,33]. These methods address different molecular questions, and their outputs require comparison with binding, target-engagement, exposure, and efficacy measurements.

Immunosuppressive tumors illustrate why these molecular questions depend on cellular context. Clinical and mechanistic studies of programmed cell death protein 1 (PD-1) and programmed death-ligand 1 (PD-L1) linked checkpoint responses to the surrounding immune state [34,35,36,37]. Studies of transforming growth factor beta (TGF-β) implicated stromal remodeling and immune exclusion in resistance [38,39,40,41]. A pathway’s association with resistance does not specify which cell compartment or therapeutic modality offers a suitable intervention. Other studies identified complementary mechanisms involving fibroblast-derived chemokines and suppressive myeloid signaling [42,43,44,45,46,47]. Vascular normalization was examined in relation to immune trafficking and drug exposure [48,49], while VEGF-A also modulated inhibitory checkpoints on T cells [50]. Adenosine signaling limited effector-cell function [51,52,53]. These mechanisms operate in different compartments and impose different requirements on drug access and activity. Their diversity supports evaluating each proposed intervention in its intended biological and pharmacological context [54,55,56].

**Figure 1:**
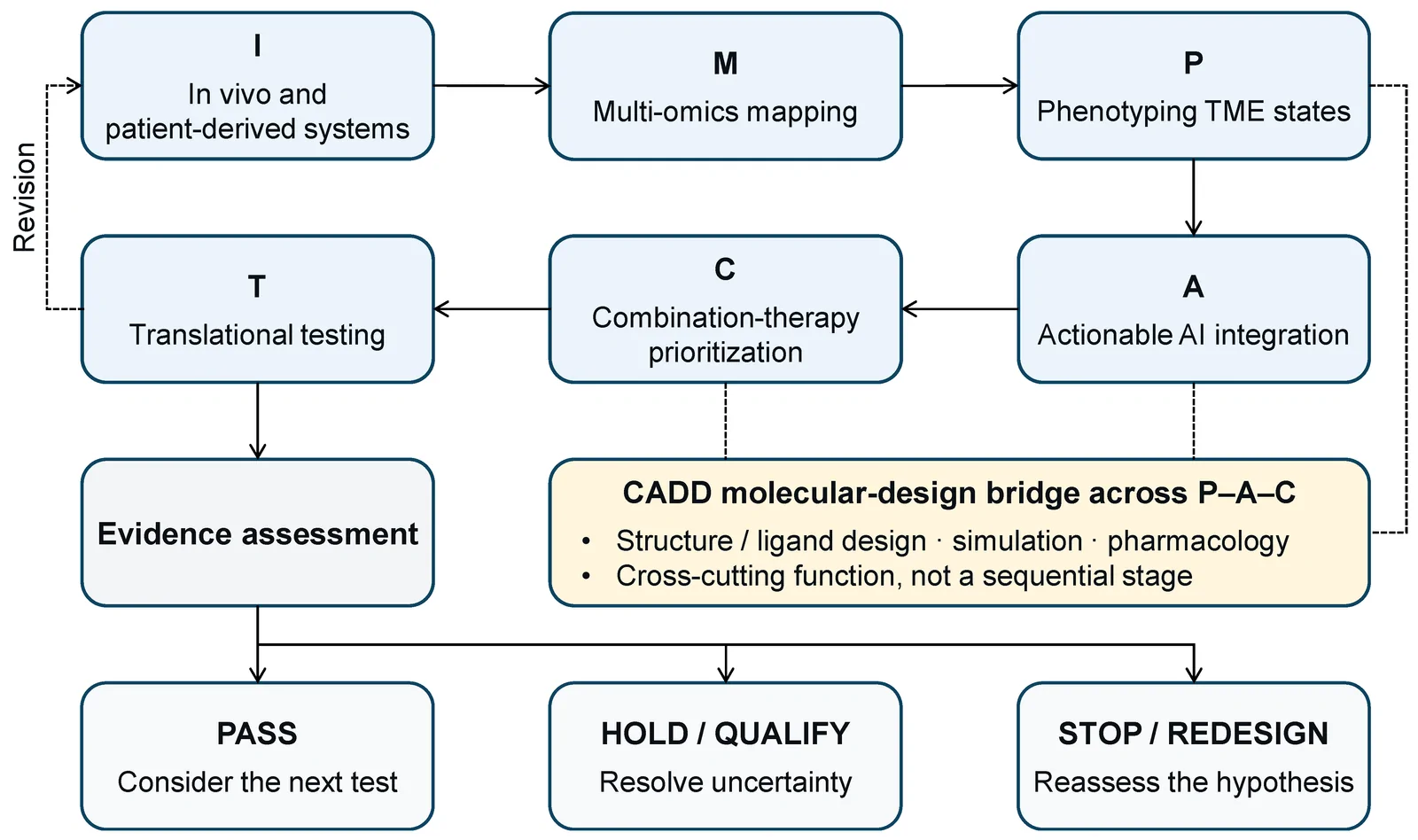
IMPACT responsibilities and the cross-cutting CADD molecular-design bridge. Solid arrows show information dependencies, not biological causation or a mandatory sequence. Dotted connectors without arrowheads link CADD to P, A, and C. The dashed return arrow shows evidence-driven revision. The branch from translational testing distinguishes PASS, HOLD/QUALIFY, and STOP/REDESIGN. These are conceptual review categories, not validated decision thresholds. C remains Combination-therapy prioritization (abbreviations: IMPACT, in vivo and patient-derived systems, multi-omics mapping, phenotyping of TME states, actionable AI integration, combination-therapy prioritization, and translational testing; TME, tumor microenvironment; AI, artificial intelligence; CADD, computer-aided drug design).

The central problem is the connection between a measured TME state and a molecule intended to change it. Spatial profiling and artificial intelligence (AI) can identify patterns associated with a state or outcome. CADD requires a specified target, a relevant structure or ligand series, and a compatible modality. This review examines how biological and computational findings inform those design choices and how experimental evidence supports each subsequent claim. We use the mnemonic IMPACT to organize this synthesis around six connected responsibilities: in vivo and patient-derived systems (I), multi-omics mapping (M), phenotyping of TME states (P), actionable AI integration (A), combination-therapy prioritization (C), and translational testing with feedback (T). Actionable AI refers to decision support for a defined experimental question. CADD contributes across phenotyping, AI integration, and combination prioritization. Figure 1 shows these connections and the evidence needed to revisit an earlier assumption.

IMPACT makes explicit how a measured state is connected to a target, modality, molecular hypothesis, and validation requirement. A spatial signature of T-cell exclusion may implicate TGF-β without selecting between an extracellular ligand trap and an intracellular receptor-kinase inhibitor. These modalities require different structural models, engagement assays, and exposure measurements. The framework records such unresolved choices; its contribution is an organized synthesis of established methods, and improved drug-development outcomes have not been demonstrated.

The remainder of this review is organized as follows: Section 2 describes literature selection and evidence classification, and Section 3 examines the biological inputs to molecular design. Section 4 reviews CADD methods and documented applications; Section 5 and Section 6 address AI interpretation, combination therapy, and translational validation. Section 7 considers evaluation and evidence records, Section 8 discusses research gaps and limitations, and Section 9 presents the conclusions. Extended evidence maps and methodological details appear in the Supplementary Material.

## 2. Review Design and Evidence Classification

### 2.1. Literature Identification and Review Boundaries

We conducted a targeted critical review of literature identified through 15 October 2026 using PubMed, publisher records, and citation chaining. The search covered patient-derived models, TME characterization, molecular design, simulation, pharmacology, and AI-supported interpretation. It included in situ structure determination, three-dimensional generative design, stromal delivery, and targeted protein degradation. Foundational studies were retained to explain the methods and biological concepts underlying these developments.

Studies were selected according to their relevance to a solid-tumor mechanism, patient-derived perturbation, computational method, documented molecular-design application, or contextual review. Selection favored identifiable targets or methods, clearly stated model systems, and interpretable validation. Primary studies supported discovery and efficacy claims; reviews supplied broader context. When a publication supported several claims, each claim was classified separately. This purposive approach was susceptible to publication and selection bias.

The three evidence-classification axes are evidence relationship, model system, and validation maturity, while evidence role and IMPACT responsibility are coded separately. Table 1 distinguishes a source’s role, inferential relationship, model system, validation maturity, and IMPACT function. Supplementary Tables S1 and S2 provide the review comparison and representative evidence map. Supplementary Sections S2–S4 document search concepts, coding fields, and reference-to-claim mapping. The synthesis was qualitative and did not use an exhaustive systematic-review or meta-analytic protocol.

### 2.2. Evidence Roles, Inferential Boundaries, and IMPACT Coding

The evidence classification separates the purpose of a citation from the strength and scope of its result. Evidence role records why a source was included, and inferential relationship distinguishes observational, perturbational, and prospective interventional findings. Model system identifies where the result was obtained; validation maturity describes replication or external testing. A well-validated predictor can remain observational, and a controlled perturbation can establish an effect within an organoid without establishing that effect in patients.

We assigned high (H), moderate (M), and low (L) capability labels to specific questions about each model class. H indicated a defining capability, M indicated partial capability requiring additional engineering or contextual support, and L indicated an absent or substantially restricted capability in a standard implementation. Standard patient-derived xenografts (PDXs) permit systemic exposure studies but lack an intact human immune system; immune co-culture can improve immune representation in patient-derived organoids (PDOs) without providing systemic pharmacokinetic (PK) modeling [5,6,56,57]. These labels were interpretive assignments rather than measured scores, and no inter-rater reliability estimate was available.

Evidence appraisal followed the type of claim. Computational studies were examined for leakage, external validation, calibration, physical validity, receptor preparation, and scaffold-, target-, or time-aware evaluation [16,17,22,58,59,60,61]. Simulation findings were considered in relation to sampling and uncertainty [24,27,29,62,63]. Biological and clinical findings were interpreted through target engagement, exposure, model fidelity, safety, and study design. These considerations supported qualitative appraisal rather than a validated risk-of-bias score.

### 2.3. Scope Relative to Existing Reviews

Previous reviews examined multimodal oncology, patient-derived models, molecular design, and combination prediction. Target-focused and subtype-focused CADD reviews synthesized lysine-specific demethylase 1 (LSD1) inhibitor discovery [64] and applications across breast-cancer subtypes [65]. This review focuses on the connections between TME-state evidence, modality-specific molecular design, and pharmacological validation. Its biological and AI sections explain the inputs and limits of molecular design; Supplementary Table S1 and Figure S1 place this scope alongside related reviews.

## 3. IMPACT Framework and Biological Evidence Layers

Biological evidence determines the starting conditions for molecular design. Patient-derived models define which responses can be tested, while multi-omics and spatial profiling identify candidate mechanisms and their cellular locations. Phenotyping connects those mechanisms to a proposed molecular target. The following sections examine the contribution and limitations of each input within the framework introduced in Figure 1.

### 3.1. In Vivo and Patient-Derived Model Systems

PDX models preserve selected patient-tumor features and allow in vivo treatment testing [6,9,57,66]. Their standard implementation uses immunodeficient hosts, and murine stromal replacement and serial passage can alter the original tissue context. PDOs, explants, and tumor fragments provide complementary opportunities for perturbing human tumor material [7,67,68,69,70]. Organoid biobanks and paired in vitro/in vivo studies demonstrated both the potential of these platforms and substantial biological heterogeneity [8,71,72]. Each model supports particular experimental questions rather than complete reproduction of the human TME. Jung et al. [73] established a colorectal liver-metastasis PDX from one patient and tested chemotherapy in immunodeficient mice. The model preserved selected tumor characteristics and enabled controlled drug-response experiments. Its single-donor origin and host immune deficiency defined the scope of the result; the study supported a preclinical testing platform rather than a demonstration of patient-specific treatment benefit.

The appropriate model depends on the response being studied. PDO studies reported treatment-response concordance in selected gastrointestinal cohorts [74,75]. Ooft et al. found that prediction depended on the regimen: organoids predicted irinotecan response but not fluorouracil–oxaliplatin outcomes [74]. Autologous organoid–lymphocyte co-cultures enabled testing of tumor-reactive T-cell killing [58]. Immune and stromal preservation, experimental duration, clonality, and systemic pharmacokinetics/pharmacodynamics (PK/PD) differ across organoids, explants, and xenografts [7,57]. Comparing complementary models helps distinguish findings that recur across systems from findings that depend on a particular model feature. Esposito et al. [76] combined colorectal-cancer organoids with autologous immune components to examine checkpoint-inhibitor resistance. They assessed marker signatures in an independent specimen cohort and used gene knockout in resistant organoids to test a proposed resistance mechanism. This example linked tissue-associated markers with functional testing in a patient-derived system, while prospective treatment selection remained a separate clinical question.

**Figure 2:**
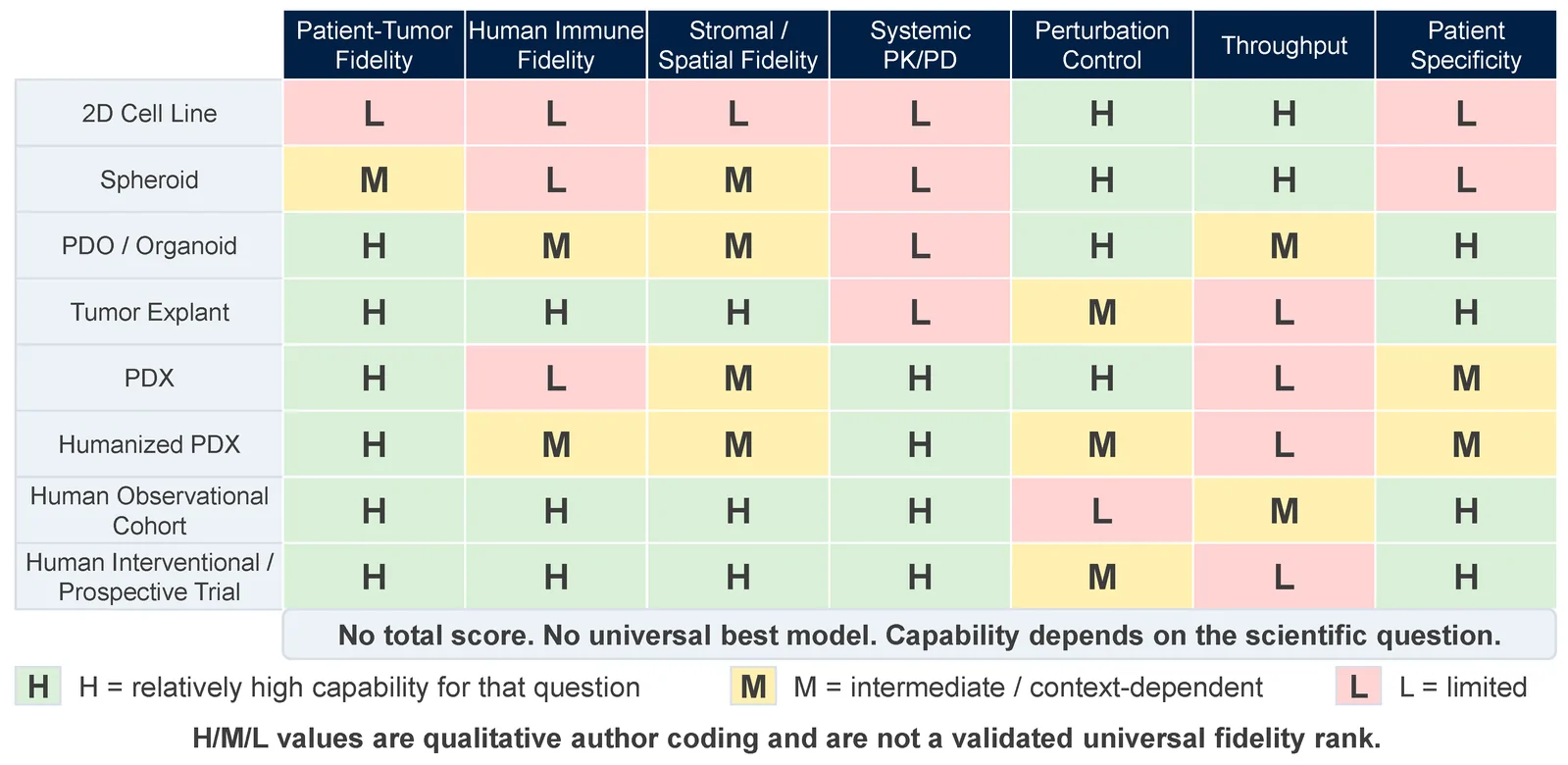
Question-specific capabilities of model systems. H indicates a defining capability of the stated model class; M indicates partial or implementation-dependent capability; L indicates absent or substantially restricted capability in a standard implementation. Ratings follow the rubric in Section 2.2 and the model limitations in Table 2. They are qualitative judgments without a total score or validated ranking. Human observational cohorts and interventional trials support different inferential claims (abbreviations: PDO, patient-derived organoid; PDX, patient-derived xenograft; PK/PD, pharmacokinetics/pharmacodynamics; 2D, two-dimensional).

Figure 2 compares model capabilities for specific questions, and Table 2 summarizes the corresponding strengths and limitations. The comparison identifies which claims each model can support. It avoids assigning an overall fidelity rank because preservation of one feature does not establish preservation of immune, stromal, spatial, and systemic behavior together.

### 3.2. Multi-Omics Mapping as a Traceable Data Asset

Multi-omics and spatial measurements identify candidate mechanisms within their cellular context. Relevant data include genomic, transcriptomic, chromatin, protein, phosphoprotein, imaging, treatment, and exposure measurements [77,78,79,80]. Spatial and single-cell studies identified local neighborhoods that bulk abundance measurements did not adequately resolve [11,81,82]. Studies of tertiary lymphoid structures found associations between immune organization and treatment response [83,84]. Such observations support phenotype definition, while a causal interpretation requires perturbational evidence. Reviews of spatial biomarkers and spatial omics examined how tissue organization could inform immunotherapy assessment [85,86], while computational immunogenomics addressed bulk and single-cell sequencing analysis [87]. Greenwald et al. [88] identified time-dependent response associations in a longitudinal breast-cancer cohort. Chen et al. [89] described lung-cancer stem-immunity hubs associated with PD-1-blockade outcomes. These studies supported spatial and temporal phenotype definition, with predictions interpreted according to sampling time and cohort design.

A phenotype becomes useful for molecular design when its target can be traced to an exact molecular entity. Relevant records include the target isoform or complex, post-translational state, structure provenance, pocket or interface annotation, compound stereochemistry, assay conditions, and exposure context. Missing descriptors can make an apparently precise state-to-target association chemically ambiguous. Keeping these records together allows a structural or pharmacological assumption to be checked against the biological observation that motivated it. Integrating these measurements introduces correlated predictors, missing modalities, and heterogeneous patient populations. Multi-Omics Factor Analysis (MOFA) modeled shared and modality-specific variation while retaining interpretable relationships across data types [90]. A latent representation can summarize those relationships without identifying a causal target or demonstrating therapeutic activity. Its usefulness for CADD depends on whether the retained biological meaning supports a specific molecular hypothesis and an experimental test.

Supplementary Figure S2 and Table S3 summarize the measurement types, computational representations, and common failure modes. For CADD, the central records connect a target and structural state to a compartment, compound, assay, and exposure. These links provide the context needed to interpret the molecular hypothesis. Studies of single-cell foundation models, including the single-cell generative pretrained transformer (scGPT), reported applications in cell representation, annotation, batch integration, perturbation prediction, and gene-network inference [91,92]. The reported tasks and validation settings differed across studies. Application to a new tumor system still requires appropriate calibration, assay provenance, and experimental assessment. These models supply representations that inform a hypothesis; their outputs alone do not establish the proposed mechanism or treatment effect.

### 3.3. Phenotyping Immunosuppressive TME States

An immunosuppressive TME comprises interacting mechanisms rather than an isolated list of biomarkers. Checkpoint activity depends on receptor–ligand interactions and the state and location of effector cells [34,36,37,93]. Studies of TGF-β receptor (TGFBR) and SMAD-family signaling linked fibroblast activation and matrix remodeling to immune exclusion in context-dependent settings [38,39,40,41]. These observations connect a spatial phenotype to candidate mechanisms while leaving the intervention’s target compartment and modality unresolved. In pancreatic-cancer models, targeting the cancer-associated fibroblast (CAF)-derived C-X-C motif chemokine ligand 12 (CXCL12) pathway through its receptor, C-X-C motif chemokine receptor 4 (CXCR4), increased T-cell access and supported anti-PD-L1 activity [42]. Colony-stimulating factor 1 (CSF1), its receptor (CSF1R), and phosphoinositide 3-kinase gamma (PI3Kγ) were implicated in suppressive myeloid programs [44,45,46,47]. Vascular endothelial growth factor (VEGF) signaling connected vascular and immune regulation [48,49,50]. Adenosine generated through cluster of differentiation 39 (CD39) and cluster of differentiation 73 (CD73) acted through adenosine A2 receptors, including the A2A receptor (A2AR), to suppress immune function [51,52,53].

Single-cell studies resolved stromal populations with different functional roles. Elyada et al. [43] identified antigen-presenting CAFs in pancreatic cancer, and Lambrechts et al. [94] mapped stromal diversity in lung tumors. Kieffer et al. [95] linked specific CAF subsets to regulatory T-cell interactions and immunotherapy resistance. Jerby-Arnon et al. [96] connected a melanoma-cell exclusion program to clinical response and tested cyclin-dependent kinase 4/6 (CDK4/6) inhibition with immunotherapy in mice. Together, these studies linked state characterization with different levels of functional evidence. A useful state-to-target description connects a reproducible phenotype to a specified mechanism and a perturbation capable of testing it. Agreement across assays strengthens the description of the phenotype. Establishing that a drug reaches and modulates the proposed target requires additional measurements. An exclusion signature can recur across samples while leaving the relevant cell compartment and therapeutic modality uncertain.

**Figure 3:**
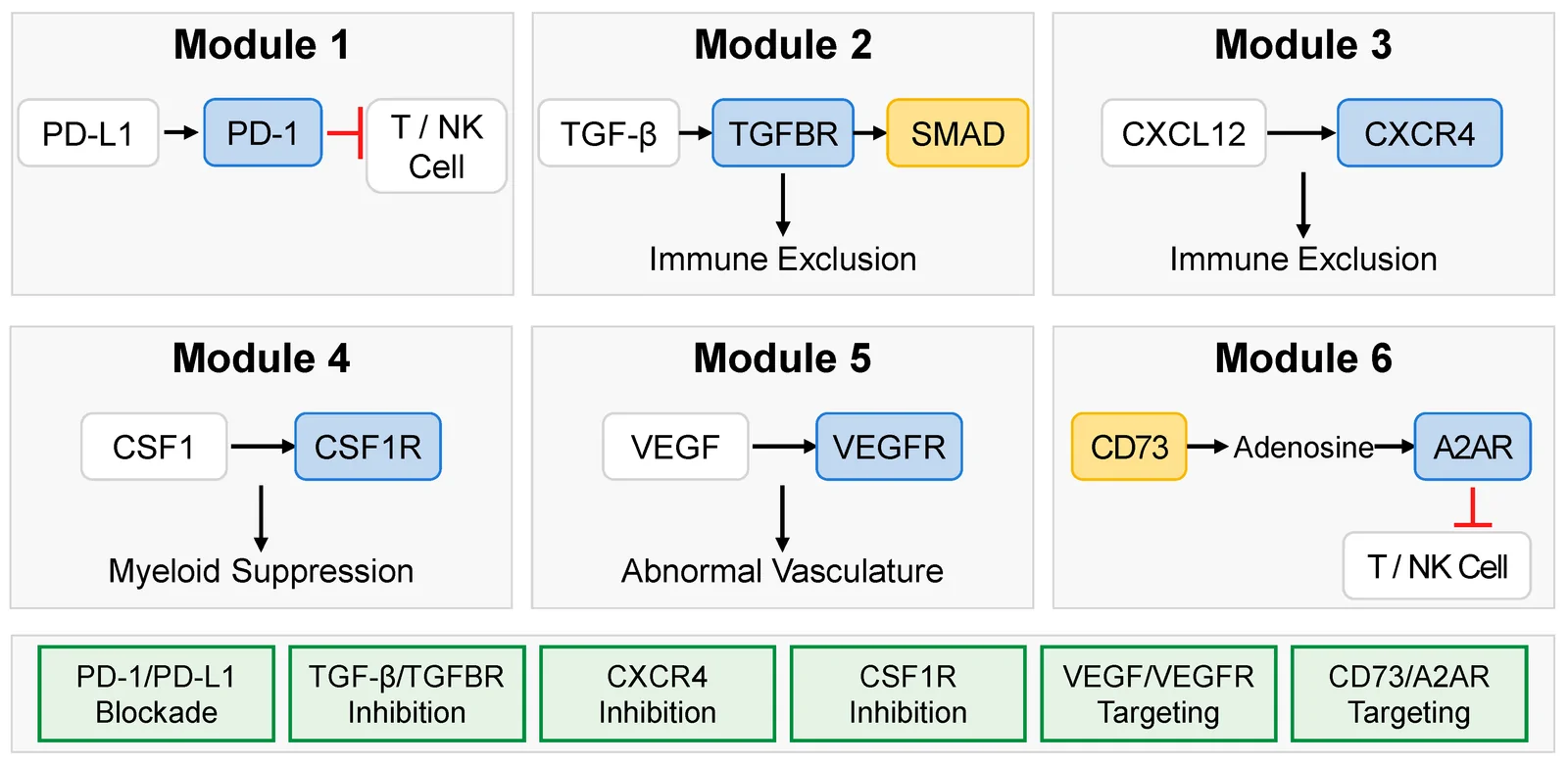
Molecular pharmacology of an immunosuppressive TME. The authors drew this schematic in Microsoft PowerPoint. The modules depict PD-1/PD-L1 checkpoint signaling, TGF-β–TGFBR–SMAD signaling, CXCL12–CXCR4 signaling, CSF1–CSF1R signaling, VEGF–VEGFR signaling, and CD73/adenosine–A2AR signaling. Blue boxes denote receptors, yellow boxes highlight other molecular mediators, and green boxes indicate potential intervention strategies. Arrowheads indicate the stated signaling or process direction, and blunt-ended lines indicate inhibition. Intervention labels identify mechanism-based hypotheses without implying demonstrated clinical benefit (abbreviations: TME, tumor microenvironment; PD-1, programmed cell death protein 1; PD-L1, programmed death-ligand 1; TGF-β, transforming growth factor beta; TGFBR, TGF-β receptor; SMAD, SMAD-family signal transducer; CXCL12, C-X-C motif chemokine ligand 12; CXCR4, C-X-C motif chemokine receptor 4; CSF1, colony-stimulating factor 1; CSF1R, CSF1 receptor; VEGF, vascular endothelial growth factor; VEGFR, VEGF receptor; CD73, cluster of differentiation 73; A2AR, adenosine A2A receptor; NK, natural killer).

Figure 3 maps representative immunosuppressive pathways to potential intervention classes, and Table 3 summarizes the corresponding molecular signals and pharmacological questions. The figure was drawn by the authors in Microsoft PowerPoint. These summaries connect biological mechanisms to testable hypotheses; clinical efficacy requires evidence beyond pathway plausibility.

Discrete labels make TME states easier to compare, but they can conceal variation within a tumor. Pan-cancer classifications and single-cell analyses resolved complementary aspects of tumor and immune heterogeneity [12,13,97]. Their interpretation depends on sampling, cell-state definitions, and assay context. A representation is useful for molecular design when it preserves the target, compartment, and structural or ligand context needed to specify an intervention. Targets with limited conventional binding sites may require allosteric, covalent, degrader, interface-directed, nucleic-acid, or indirect strategies [98]. Each modality introduces a different design problem. Antisense approaches target ribonucleic acid (RNA), while triplex-forming antigene oligonucleotides require recognition of compatible duplex deoxyribonucleic acid (DNA) and access to the nucleus [99]. The methodological basis for these approaches informs modality selection without establishing efficacy in an immunosuppressive TME.

## 4. Computer-Aided Drug Design Within the IMPACT Loop

CADD connects a target hypothesis to candidate molecules and testable interaction mechanisms. The literature supports distinct roles for structure qualification, virtual screening, ligand-based prioritization, molecular simulation, and developability assessment. This section compares those roles and adds documented applications to distinguish computational contribution from subsequent biological validation.

### 4.1. From TME Phenotype to Molecular Targetability

A molecular-design problem specifies the target isoform or complex, the relevant cell and compartment, and the intended modality. An extracellular ligand trap, membrane-receptor antagonist, and intracellular degrader can address related pathways but require different structures, access routes, and engagement assays. Expression or pathway enrichment alone does not resolve these design choices. Structural tractability depends on both the target state and the intended modality. Shallow pockets, transient interfaces, cryptic sites, and disordered regions may be approached through allosteric, covalent, fragment-based, degrader, or indirect strategies [98]. A visible pocket alone does not establish that a useful ligand series can be developed against it. The design question concerns whether the relevant molecular state can be perturbed at the required cellular location and exposure.

Experimental structures are most informative when they represent a pharmacologically relevant state, while predicted models retain uncertainty around pockets, interfaces, and flexible regions [14,15]. In situ structural methods add information about native cellular context. Wagner et al. [100] combined cryogenic focused ion-beam preparation, cryo-electron tomography, and subtomogram analysis to visualize the chaperonin cycle inside cells. That study provided a methodological example of native-state analysis. Application to tumor tissue depends on sample thickness, target abundance, structural heterogeneity, and attainable resolution. Spatial proteomics supplies information about where a target is expressed and which cells surround it. Multiplexed tissue studies mapped protein expression and cellular neighborhoods [11,82]. These measurements can identify a relevant target compartment without resolving an atomic binding pocket or conformational ensemble. In situ structural data can constrain the molecular state when sufficient resolution is available. Combining these sources helps select the state to model, with target-specific structural validation remaining necessary.

### 4.2. Structure-Based and Ligand-Based Design, Docking, and Virtual Screening

Structure-based design requires a binding site and receptor state appropriate to the molecular question. Classical studies evaluated docking search and scoring accuracy [16,17,101,102]. CASF-2016 distinguished scoring, ranking, docking, and screening tasks [18]; DUD-E supplied ligand–decoy benchmark sets [61]; and Glide studies measured screening enrichment [103]. ZINC provided purchasable compounds prepared for virtual screening [104]. AutoDock4 incorporated selective receptor flexibility [105]. These contributions inform different choices in receptor preparation, compound selection, and performance evaluation. Deep-learning approaches extended the available strategies for pose generation and scoring. GNINA, DiffDock, and geometry-based methods addressed distinct aspects of these tasks [20,21,106,107]. CrossDocked and PoseBusters analyses identified redundancy, physical invalidity, and limited generalization as sources of misleading benchmark performance [22,23]. A low positional error or favorable ranking score does not establish affinity or biological activity. Experimental and orthogonal computational checks are needed to assess the resulting pose hypothesis.

Ligand-based methods provide a complementary option when structural information is incomplete. Quantitative structure–activity relationship (QSAR) models, pharmacophores, similarity searches, and learned molecular representations prioritize compounds within a defined chemical and assay domain [59,60,62,108]. Large-scale ChEMBL comparisons found that target diversity, chemical redundancy, and evaluation protocol influenced apparent performance [109]. Scaffold-, time-, and project-aware validation test forms of generalization that random splitting can overlook. Three-dimensional generative methods extend molecular design beyond pose selection for existing ligands. DiffSBDD used a rotation- and translation-equivariant model to generate pocket-conditioned ligands and support constrained molecular redesign [110]. DiffGui combined atom and bond generation with property guidance and reported experimental testing of selected generated compounds [111]. These studies addressed ligand generation, while DiffDock and EquiBind addressed poses of supplied ligands [21,106]. Their reported results did not establish general efficacy against TME protein interfaces.

Protein-binder generation addresses broad interfaces through a different molecular modality. RFdiffusion produced experimentally tested binders to targets including PD-L1 [112]. Binding to PD-L1 does not by itself establish checkpoint blockade or antitumor activity. Epitope accessibility, glycosylation, selectivity, competition, and compartment-specific exposure remain relevant to that interpretation. GuacaMol and MOSES provided benchmarks for generated small molecules [113,114]. Reinforcement-learning methods optimized generation toward specified properties [115], while medicinal-chemistry commentary examined how to assess claims of practical value [116]. Documented applications show how computation contributed to discovery and where experimental support entered. Congreve et al. used structure-based methods to develop 1,2,4-triazine A2AR antagonists [117]. A separate study of AZD4635 combined receptor structural characterization with binding, signaling, and preclinical immune-function experiments [118]. RFdiffusion generated candidate PD-L1-binding surfaces that were tested experimentally [112]. These examples support specific design and binding contributions; checkpoint inhibition and patient benefit require additional functional and clinical evidence. These examples are summarized in Table 4.

**Figure 4:**
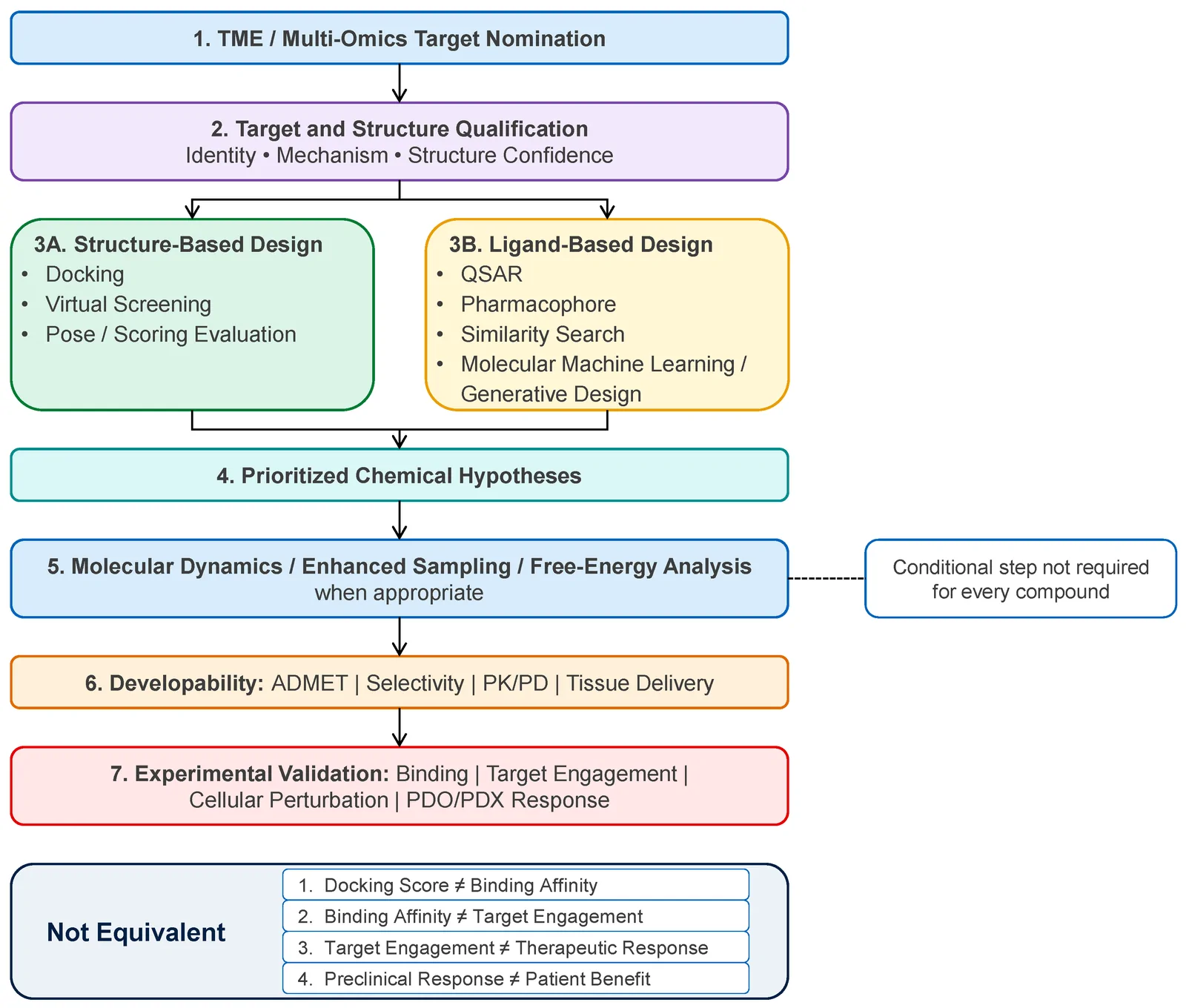
CADD methods connecting TME-informed target nomination to testable molecular hypotheses. Arrows indicate workflow or evidence progression. Simulation, free-energy analysis, developability assessment, and pharmacology address distinct questions selected for the candidate and modality. Docking score, binding affinity, target engagement, therapeutic response, and patient benefit require separate support (abbreviations: CADD, computer-aided drug design; TME, tumor microenvironment; ADMET, absorption, distribution, metabolism, excretion, and toxicity; PK/PD, pharmacokinetics/pharmacodynamics; QSAR, quantitative structure–activity relationship; PDO, patient-derived organoid; PDX, patient-derived xenograft).

Figure 4 summarizes these distinct CADD layers as a progression from TME-informed target nomination to experimentally falsifiable chemical hypotheses. Table 5 compares the molecular questions addressed by these methods with their validity conditions and limits. A more elaborate calculation does not automatically support a stronger biological claim. Each result is interpreted according to the quantity it estimates and the experimental evidence available for that quantity.

Applying CADD to a TME mechanism requires a modality-specific design question. Table 6 connects representative states to structural requirements, target-engagement measurements, and exposure constraints. The mechanistic citations support the biological pathway, while the computational literature supports the scope and limitations of the design method. These evidence types cover different parts of the proposed intervention.

The design relationships in Table 6 identify opportunities and unresolved requirements rather than completed routes to clinical benefit. A review of Chinese materia medica research documented applications of docking, simulation, and network-based prioritization [120]. Such applications illustrate computational scope. A proposed compound–target relationship still requires verification of molecular identity, binding, and activity in the intended TME context.

### 4.3. Molecular Simulation and Emerging Therapeutic Modalities

Docking proposes a pose; molecular simulation examines its compatibility with a specified molecular state. Receptor flexibility, solvent organization, protonation, ligand ionization, cofactors, and membranes can alter the predicted interaction. An interpretable MD analysis specifies the starting ensemble, force field, replication strategy, and convergence criterion [24,25,26]. Retention of a pose in a trajectory supports conformational compatibility within the modeled system. It does not directly measure binding affinity. TME conditions become simulation inputs when they can be represented as molecular or pharmacological variables. Acidity can alter protonation, hypoxia can affect redox or cofactor states, and membrane composition can shift receptor conformations. Fibrosis, perfusion, and vascular dysfunction are addressed more directly through transport or exposure models [48,49,121]. The model’s mechanistic scope determines how each biological condition can be represented and what interpretation its output can support.

Alchemical free-energy calculations estimate energetic differences between specified molecular states. Their interpretation depends on ligand-series suitability, sampling, charge treatment, and uncertainty [28,63,122]. Prospective applications demonstrated utility in active discovery projects [29,30], while an absolute-affinity benchmark examined BRD4 inhibitors [123]. A recent review synthesized developments across these approaches [27]. Ross et al. [124] improved predictions for transformations involving buried water by combining molecular dynamics with grand canonical Monte Carlo sampling. These results inform binding-energy estimates within the tested systems. QM/MM addresses mechanisms that require explicit treatment of electronic changes. Relevant applications include covalent bond formation, proton transfer, metal coordination, and reaction energetics [31,32,33,125]. The quantum region, boundary treatment, electronic state, reaction coordinate, and conformational sampling define the calculation. Its value depends on whether these features answer the molecular question. Calculated reaction energetics do not independently establish selectivity, exposure, or safety.

A candidate’s affinity becomes therapeutically relevant only when adequate pharmacological exposure is achieved. Predictions of absorption, distribution, metabolism, excretion, and toxicity (ADMET) can support early selection within their applicability domains [126,127,128,129]. Prediction platforms expanded endpoint coverage [130,131], and industrial analyses identified exposure, target engagement, pharmacology, and safety as recurring development constraints [132,133,134,135]. Candidate assessment connects predicted properties with measured free concentration, cellular engagement, off-target activity, and tolerability. Solid tumors impose additional barriers between systemic exposure and local drug availability. Perfusion, interstitial transport, fibrosis, cellular uptake, and spatial compartmentalization affect access to the intended target [121,136,137]. Nanoparticle and precision-delivery studies showed that systemic administration did not ensure uniform tumor entry or distribution [138,139]. These findings make delivery and PK/PD part of the combination hypothesis. Insufficient target-compartment exposure limits the interpretation of favorable molecular scores or in vitro synergy.

CAF-directed delivery links molecular formulation to stromal access. Liu et al. [140] reported a fibroblast activation protein (FAP)-responsive nanocarrier containing the fibronectin-derived peptide FNIII14. The formulation modulated CAF-associated matrix production and improved penetration in preclinical tumor models. The study illustrates a preclinical delivery strategy rather than docking-based molecular discovery. Relevant design variables include particle properties, ligand presentation, release kinetics, stromal distribution, and payload exposure. Targeted degradation adds requirements beyond binding to a single protein. Lu et al. [141] used self-assembled peptide proteolysis-targeting chimera (PROTAC) nanoparticles to degrade signal transducer and activator of transcription 3 (STAT3) and β-catenin in colorectal-cancer models. They reported changes in the immune microenvironment, without establishing macrophage-specific delivery or clinical myeloid reprogramming. PROTAC design depends on target–ligase geometry, linker behavior, cellular access, ligase availability, degradation kinetics, and selectivity.

Molecular glues recruit proteins through a different interaction mechanism. A structural study showed how lenalidomide promoted recruitment of casein kinase 1 alpha (CK1α) to cereblon (CRBN) [142]. That study established a recruitment principle outside a TME-directed therapeutic setting. Applying the principle to suppressive myeloid states requires evidence of degradation in the relevant cells, the resulting phenotype, and functional immune effects.

### 4.4. Minimum CADD Reporting and Experimental Handoff

An interpretable CADD output links a candidate’s identity to its target state, predicted interaction, uncertainty, selectivity, and exposure assumptions. The next assay tests the weakest consequential assumption: binding when the interaction is uncertain, cellular engagement when access is uncertain, or a mechanism-linked perturbation when the phenotype is ambiguous. Supplementary Table S5 records the longer implementation checklist.

Binding, target engagement, and patient-derived response answer different questions. Orthogonal biochemical measurements test interaction, cellular assays test access and engagement, rescue or rechallenge experiments test mechanism, and exposure-matched patient-derived models test response in a more relevant biological context. Evidence at one layer can motivate the next experiment without establishing its result.

## 5. AI Integration, Causal Inference, and Treatment Policy

### 5.1. Multimodal Fusion, Foundation Models, and Biological Priors

Multimodal fusion combines inputs, representations, or decisions [2,143,144,145]. In TME-oriented design, its contribution is evaluated against a defined task, such as state classification, target nomination, or experiment prioritization. Graph networks, pathology encoders, transformers, and foundation models offer different representations; comparisons with strong tabular, pathway-based, or unimodal baselines clarify whether the added complexity helps that task. Validation depends on identifying the appropriate unit of biological independence. Cells, image tiles, organoid wells, serial samples, and PDX animals from one donor do not constitute independent patients. Missing-modality handling, calibration, uncertainty estimation, and explanation also affect the interpretation of model performance [2,143,146]. Distinguishing biological from technical replication prevents a large number of measurements from being mistaken for broad patient-level evidence.

Chemical and structural similarity introduce additional dependence in drug-design datasets. Studies of evaluation protocols and docking benchmarks showed that related scaffolds, homologous targets, replicated assay series, and similar complexes could inflate performance estimates [22,23,60,62]. Benchmark interpretation also depends on decoy-construction rules and on whether scoring, docking, ranking, or screening is being evaluated [18,61]. Scaffold-, time-, target-, and project-aware evaluations probe different aspects of generalization. Their relevance depends on whether the intended application involves new chemistry, unfamiliar targets, or prospective discovery. Prospective or temporally separated evaluation tests performance on later compounds rather than repeated interpolation within an existing chemical domain [62,147]. A new patient population, assay protocol, target state, or scaffold may fall outside the training data’s support. Confidence estimates are informative only when they reflect that shift. Numerical precision alone cannot compensate for missing evidence about generalization.

Supplementary Figure S3 and Table S4 compare the AI integration stack and model families. Their role here is to qualify biological and molecular hypotheses rather than provide an independent ranking of treatment efficacy. Editorial and review articles discussed foundation models and large language models (LLMs) for multimodal representation and cancer decision support [148,149,150]. Molecular recommendations remain constrained by target identity, compound identity, physical validity, training-domain overlap, and experimental support [23,151,152]. These constraints apply across model scales. Their assessment connects the recommendation to a specific biological and molecular question.

Biological priors can make a computational hypothesis more explicit. Pathway topology, ligand–receptor relationships, protein complexes, and structural constraints connect a predicted TME state to a proposed molecular mechanism. Within IMPACT, the relevant question is whether the prior narrows an experimentally testable hypothesis and clarifies the molecular-design handoff described in Section 4.4. Encoding a predicted interaction in a model architecture does not convert it into an experimentally established mechanism. Biological priors restrict the hypothesis space but can preserve outdated or tumor-specific assumptions. Comparisons with less constrained models can test their value. For CADD, a pathway prior becomes useful only after it is linked to a target state, accessible compartment, and mechanism-specific perturbation.

### 5.2. Prediction, Intervention, and Transportability

Accurate treatment-response prediction does not automatically identify the effect of changing treatment. Observational data reflect existing assignment decisions shaped by severity, clinician judgment, biomarkers, previous therapy, comorbidity, and institutional practice. A model can learn these selection patterns and predict outcomes without estimating what would happen under a different intervention. Equation (1) describes this predictive quantity:

*E*(*Y*∣*X*, *A* = *a*) (1)

In Equation (1), *X* denotes the measured tumor and patient state, *A* the observed treatment, and *Y* the outcome. The resulting quantity is predictive because it is conditioned on the treatment assignments actually observed in the data. It may capture clinically useful association while remaining insufficient to identify a causal treatment effect.

An interventional estimand asks what outcome would be expected if treatment were set to a specified action. This question changes the interpretation of the quantity being estimated. Equation (2) expresses that intervention-based target, whose identification requires assumptions beyond predictive accuracy:

*m_a_*(*X*) = *E*[*Y*∣*X*, *do*(*A* = *a*)] (2)

Identification of the quantity in Equation (2) requires defensible consistency, treatment support or positivity, and appropriate control of confounding [153]. Experimental evidence can supply support that an observational association lacks. A flexible AI model can improve prediction within the available data without resolving those identification requirements. Model complexity alone cannot recover an unsupported causal effect.

**Figure 5:**
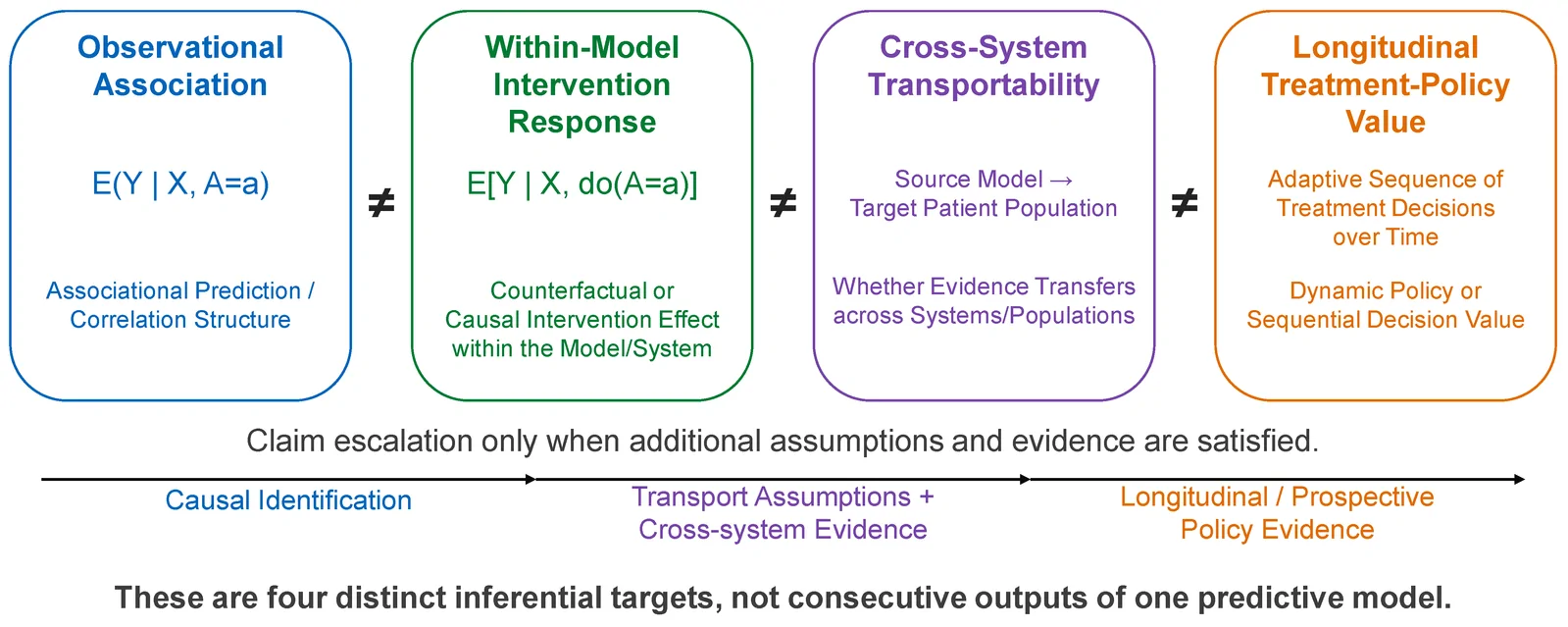
Four distinct inferential targets: observational association, within-model intervention response, cross-system transportability, and longitudinal treatment-policy value. The ≠ symbols indicate non-equivalence. The lower arrows indicate conditional escalation of claims when additional causal, transportability, and longitudinal or prospective evidence is available. They do not depict a biological causal pathway (symbols: X, measured tumor and patient state; A, treatment; Y, outcome; do(A), an intervention setting treatment A).

An effect identified within an experimental model faces an additional question of transportability [154]. PDOs, PDXs, and explants differ from patients in immune, stromal, pharmacological, and population context [7,57,66]. A credible within-model effect can fail to generalize when those differences alter the mechanism or exposure. Figure 5 separates within-model intervention response from model-to-human transportability and longitudinal treatment value.

Predictive models are used here primarily for stratification, representation, and hypothesis generation. Stronger intervention claims require perturbational evidence, while patient-level claims require evidence supporting transportability or prospective evaluation. This separation prevents improvements in predictive discrimination from being interpreted automatically as improvements in treatment decision quality.

### 5.3. Longitudinal Treatment Policies

For CADD-informed combination therapies, treatment decisions must also account for how earlier interventions affect subsequent therapeutic options. An early intervention can alter tumor burden, immune composition, toxicity, resistance, and the set of feasible later treatments. The treatment with the strongest immediate response may offer less long-term benefit if it limits subsequent options. Dynamic treatment-regime research formulated therapy as a sequence of decision rules that mapped evolving patient information to stage-specific actions [155,156,157]. Equation (3) is defined as follows:

(3) $$V^{\pi }=E\left[\sum_{t=1}^{T}u_{t}(S_{t},\;A_{t})\right]$$

In Equation (3), π denotes a treatment policy, *S_t_* the evolving patient or tumor state at decision time *t*, *A_t_* the selected action, and *u_t_* the stage-specific utility, which may include efficacy and toxicity. The equation defines a conceptual objective; it is not an estimated or validated treatment policy. Estimation requires adequate longitudinal data, treatment support, control of time-varying confounding, and clinically meaningful utility definitions.

Response prediction, treatment-effect estimation, transportability, and policy optimization answer different clinical questions. Each requires evidence suited to its intended claim. This distinction matters when AI ranks combinations because a favorable early response may be offset by toxicity, resistance, or loss of later treatment options. An interpretable ranking makes these assumptions and time horizons explicit.

## 6. Combination Therapy and Translational Validation

### 6.1. Combination-Therapy Prioritization

Combination studies are most interpretable when the partner agents address a defined TME mechanism: TGF-β-associated exclusion [38,39,41], vascular dysfunction [48], suppressive myeloid signaling [44,46], or adenosine-mediated suppression [51,52]. The relevant comparison is whether a partner improves a mechanism-linked response at compatible exposure and tolerable toxicity, not whether it raises a synergy score alone. Dose, schedule, tissue exposure, toxicity, formulation, and biomarker context can change the value of a combination. Individually plausible agents may be incompatible when their effective exposures do not overlap, toxicity accumulates, or the second target is inactive in the selected state. Reviews of anticancer drug–drug interaction prediction addressed potential changes in drug effects and toxicity [158]. These safety-oriented assessments complement efficacy-oriented synergy analysis.

Synergy metrics summarize combination responses under different reference models. Bliss independence, Loewe additivity, highest-single-agent (HSA), and zero-interaction-potency (ZIP) quantify related but distinct departures from an expected response [159,160,161,162]. A combination can appear synergistic under one metric, additive under another, or favorable only within a narrow dose range. Large combination datasets and benchmarks enabled model development while leaving substantial gaps between cell-line response and clinically useful combinations [163,164,165]. An in vitro synergy score does not establish target engagement, compatible exposure, tolerability, or durable TME reprogramming. Dose and schedule determine which intervention was actually tested. Evidence of a mechanism-linked perturbation is needed to connect the response surface to a translational claim. Patient-derived validation is especially relevant when the proposed combination depends on stromal, immune, vascular, or metabolic features absent from conventional cell lines.

**Figure 6:**
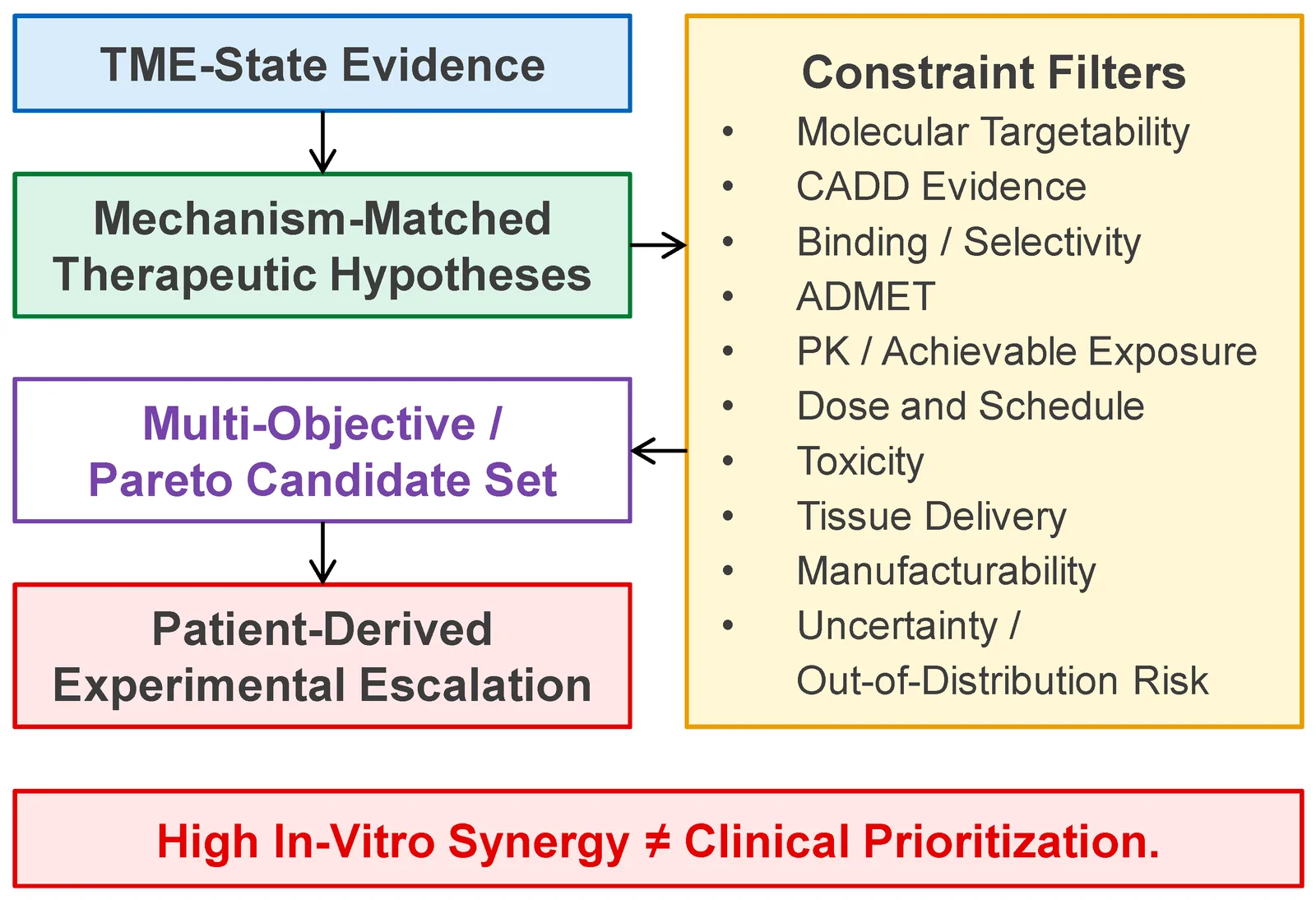
Mechanism-matched combination prioritization as a constrained pharmacology problem. Solid arrows indicate decision workflow from TME-state evidence to candidate hypotheses, CADD/developability filters, dose–schedule constraints, and a multi-objective Pareto set before patient-derived escalation. A high in vitro synergy score is not equivalent to clinical prioritization (abbreviations: TME, tumor microenvironment; CADD, computer-aided drug design; ADMET, absorption, distribution, metabolism, excretion, and toxicity; PK, pharmacokinetics).

Figure 6 summarizes combination prioritization as a constrained, multi-objective decision process rather than a single-score ranking problem. Table 7 illustrates how representative combination strategies can be evaluated by asking a mechanism-specific failure question before escalation.

### 6.2. A Retrospective Illustration from INTR@PID Lung 037

We used INTR@PID Lung 037 as a retrospective illustration of the questions organized by IMPACT. The trial was not designed to test the framework, and the analysis was conducted after its outcome was known. This application examined unresolved biological and pharmacological assumptions. It could not estimate predictive accuracy or decision benefit, nor could it determine whether the trial’s failure was preventable. The trial randomized 304 patients with treatment-naive, PD-L1-high advanced non-small-cell lung cancer (NSCLC) to bintrafusp alfa or pembrolizumab. Bintrafusp alfa combined a PD-L1 antibody with a TGF-β trap. It did not demonstrate superior progression-free or overall survival; grade 3–4 treatment-related adverse events were more frequent, and the study was stopped for futility [119]. These results established the comparative clinical outcome without identifying a single biological cause of failure.

Several explanations remained compatible with the clinical finding. The enrolled population might not have been sufficiently enriched for TGF-β-dependent exclusion, or the added pathway intervention might have offered limited incremental benefit. Toxicity or pharmacological burden could have offset efficacy, and biological heterogeneity could have diluted benefit in a subgroup. The published outcome did not resolve these alternatives. Table 8 applies the framework to these unresolved questions as a retrospective, hypothesis-generating analysis. It links the observed outcome to questions about model relevance, phenotype, target engagement, and exposure. These are questions that could be specified in a prospective study, without implying that IMPACT would have predicted the trial result.

The analysis distinguishes observed trial results from possible explanations that require testing. Patient enrichment, pathway dependence, exposure, and toxicity remain separate hypotheses. Evaluating IMPACT prospectively would require predefined criteria and comparison with an explicit alternative decision process before outcomes became available. No such evaluation was performed in this review.

### 6.3. Translational Evidence Gates and Closed-Loop Validation

**Figure 7:**
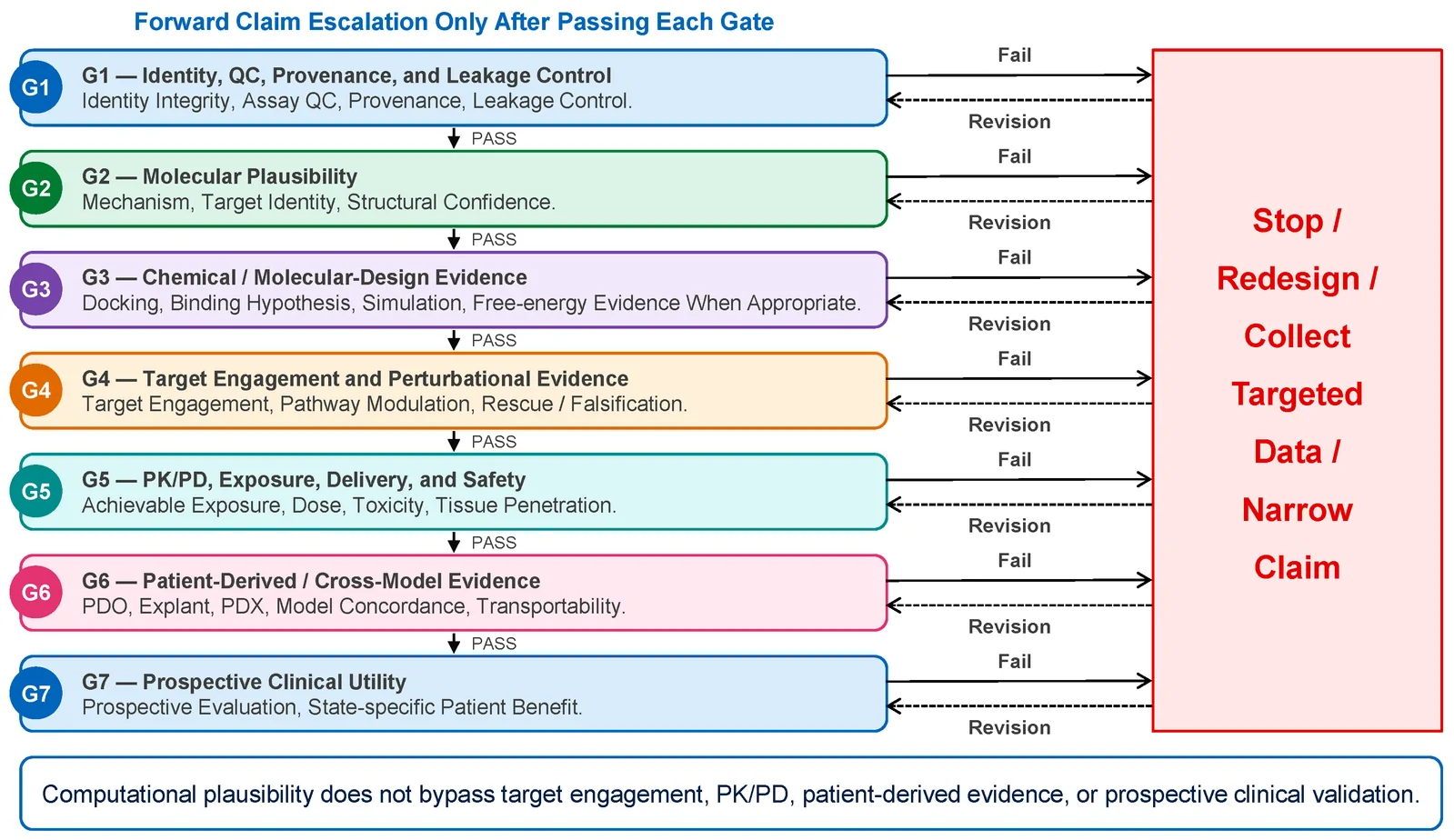
Illustrative Evidence Gates for Claim Escalation (Not a Fixed Sequence). Downward PASS arrows indicate support for considering the next claim. Rightward Fail arrows lead to stopping, redesign, targeted data collection, or claim restriction; dashed leftward Revision arrows return new evidence to the relevant gate. A non-pass result does not advance automatically. These conceptual categories do not specify validated thresholds or establish clinical utility (abbreviations: QC, quality control; PK/PD, pharmacokinetics/pharmacodynamics; PDO, patient-derived organoid; PDX, patient-derived xenograft; G1–G7, gates 1–7).

The evidence gates in Figure 7 and Table 9 summarize the claim boundaries identified across the literature. PASS means that a prespecified minimum for the intended next experiment or claim is met; HOLD/QUALIFY records remediable uncertainty; STOP/REDESIGN records evidence incompatible with the stated intended use. These are proposed categories, not empirically calibrated thresholds. The gates represent distinct evidence requirements rather than a mandatory sequence, and their assessment may be iterative or modality-dependent.

New findings can change the interpretation of an earlier target, structural state, exposure assumption, or intended population. The gates record the reason for further testing, revision, or discontinuation. Their order follows the question under evaluation and does not impose a universal linear development sequence.

## 7. Evaluation and Implementation

### 7.1. Decision-Centric Evaluation

Evaluation depends on the claim. State classifiers are assessed for discrimination, calibration, cross-assay reproducibility, and donor independence. Molecular screens are assessed for pose or enrichment performance, physical validity, and scaffold- or target-aware generalization [18,19,23]. Time- and project-aware evaluation better approximates prospective ligand design than random compound splitting [59,60,62]. Simulation evidence depends on convergence and uncertainty [28,30], ADMET estimates depend on endpoint validation and applicability domains [126,127,131], and combination ranking depends on dose–surface behavior, tolerability, and experimental confirmation [159,160,163]. Performance in one domain does not validate another. Table 10 summarizes evaluation domains according to the type of claim they are intended to support.

### 7.2. Evidence Records for Molecular Design

A usable evidence record links biological material [6,7,9,71], molecular inputs [19,28,127], and measured pharmacology [121,134,137]. Supplementary Table S5 lists the detailed artifacts. Maintaining those links allows an apparent failure to be examined at the level of target choice, chemistry, delivery, or model scope, rather than attributed to an undifferentiated computational pipeline.

## 8. Research Gaps and Limitations

### 8.1. Research Gaps

The following research gaps impacted this study:

- Matched evidence remains scarce. Few datasets contain patient-linked spatial states, target structures, compound identities, target engagement, exposure, toxicity, and outcomes. This limits assessment of whether the molecular hypothesis addresses the state measured in the same biological system.
- Perturbational and longitudinal evidence is also incomplete. Retrospective treatment associations may reflect clinical selection, while static bulk profiles can miss local or transient resistance states. Longitudinal spatial measurements and exposure-matched perturbation assays can test these unresolved links.
- Computational generalization remains a separate limitation. Benchmark analyses identified structural redundancy and chemical overlap as sources of inflated performance [19,22,23,60,62]. Inadequate sampling also weakens simulation estimates [28,30]. Prospective synthesis and binding tests provide direct evidence about performance on novel scaffolds or unfamiliar target states.
- Discordance across biochemical assays, organoids, xenografts, and patients can identify model-specific dependencies. Interpreting that discordance requires matched compounds, concentrations, target-engagement measurements, and readouts; averaging incompatible results can hide the reason for disagreement.
- Expert review remains important for structure preparation, chemical synthesis, assay design, and pharmacological interpretation. AI-assisted retrieval or orchestration can support these activities, but does not supply missing experimental evidence.

### 8.2. Limitations

This review used purposive selection rather than exhaustive screening. Screening counts and independent duplicate coding were not available, and the evidence map and capability labels reflected interpretive judgments. Operational definitions made the assignments more explicit without establishing their reproducibility. Differences among model classes, tumor states, modalities, and compounds also precluded a universal fidelity score.

CADD connects a biological phenotype to a molecular hypothesis, with experimental support required at each subsequent stage. Docking, MD, free-energy calculations, QM/MM, and AI do not provide validated substitutes for therapeutic efficacy. The retrospective trial analysis in Section 6.2 generated questions about a published outcome. It did not identify the causal reason for that outcome or test the framework’s ability to predict development success.

IMPACT is an organizational proposal whose prospective benefit has not been tested. Disease-specific instantiation would require explicit targets, structures, modalities, compounds, exposure assumptions, and intended claims. The illustrative gates cannot determine clinical treatment or replace experimental and clinical assessment.

## 9. Conclusions

The literature supports a clear boundary between observational AI-driven state prediction and CADD-generated molecular hypotheses intended for intervention. A predicted tumor state can nominate a target; a molecular design can propose how to engage it. Neither establishes the effect of changing treatment. Binding, cellular engagement, exposure, model-specific response, and patient benefit require their own evidence. Adenosine-receptor antagonist discovery and generative PD-L1-binder design demonstrated different computational contributions supported by distinct experiments. Native-state structural studies, stromal-delivery systems, and protein-degradation studies expanded the available approaches to molecular intervention. Their translation to a specified TME state requires experiments matched to the target, modality, compartment, and intended claim.

IMPACT organizes these connections through the six responsibilities defined in the Introduction. CADD contributes across phenotyping, AI integration, and combination prioritization, while experimental feedback can revise an earlier assumption. The bintrafusp alfa trial served as a retrospective illustration of unresolved evidence requirements. The framework’s prospective value for improving decisions remains untested. Progress will depend on linking molecular design to traceable experimental evidence in the intended TME state. This requires matched biological models, qualified targets and structures, measured pharmacology, and prospective testing of the resulting intervention hypotheses.

## Figures and Tables

**Table 1:** Evidence-role framework, three-axis evidence-classification scheme, and independent IMPACT functional coding.

Classification Dimension	Operational Levels	What It Supports	What It Must Not Imply
Evidence role	Direct solid-tumor translational; adjacent oncology; foundational/transferable methodology; review/synthesis	Why a study is used in this review	Methodological relevance does not constitute evidence of anticancer efficacy
Evidence relationship	Observational/predictive; perturbational; prospective interventional; N/A	Strength and type of within-system mechanistic or causal inference	Perturbation within a model does not automatically establish a causal effect in patients
Model system	Cell line/spheroid; PDO; explant; PDX; humanized PDX; human observational; human interventional	Biological scope within which a claim is interpreted	Model systems are question-specific categories rather than a universal fidelity hierarchy
Validation maturity	Internal; orthogonal/external; prospective/co-clinical; N/A	Replication, reliability, and translational maturity	External validation alone does not establish causal transportability
IMPACT responsibility	I/M/P/A/C/T, multi-label; CADD bridge recorded separately as cross-cutting	Where the evidence functions within IMPACT	Functional assignment is not an evidence grade, and CADD is not the formal C

Abbreviations: CADD, computer-aided drug design; IMPACT, in vivo and patient-derived systems, multi-omics mapping, phenotyping of TME states, actionable AI integration, combination-therapy prioritization, and translational testing; N/A, not applicable; PDO, patient-derived organoid; PDX, patient-derived xenograft.

**Table 2:** Question-specific capabilities and limitations of model systems used in TME-centered translational AI.

Model System	Preserved Strengths	Primary Limitations	Best-Supported Claim in IMPACT
2D/cell lines	Perturbation control; throughput	Weak spatial, immune, and stromal fidelity	Mechanistic prescreening
Spheroids	3D growth; scalable perturbation	Limited human immune/stromal diversity	Combination prescreening
PDO/organoids	Patient tumor genotype; controlled treatment	Variable TME retention; culture selection	Patient-specific tumor response; selected TME perturbation
Tumor fragments/ explants	Native spatial, immune, and stromal context	Short viable window; limited expansion	Short-term human-tissue perturbation
PDX	Patient tumor architecture; in vivo PK/PD	Immunodeficient host; murine stromal replacement	Tumor-intrinsic response and in vivo pharmacology
Humanized PDX	Adds selected human immune components	Variable reconstitution; human leukocyte antigen (HLA) and graft-versus-host disease (GVHD) constraints	Immune-oncology hypotheses with explicit model caveats
Human observational cohort	Target population; real clinical context; longitudinal associations	Confounding, treatment-selection bias, missingness, limited perturbation control	Clinical association; prognostic or predictive biomarker evidence
Human interventional/ prospective trial	Target population; protocolized intervention; prospective outcomes	Eligibility constraints, sample size, workflow effects, and limited mechanistic depth	Prospective intervention effect or clinical utility within the trial design

Abbreviations: IMPACT, in vivo and patient-derived systems, multi-omics mapping, phenotyping of TME states, actionable AI integration, combination-therapy prioritization, and translational testing; TME, tumor microenvironment; PDO, patient-derived organoid; PDX, patient-derived xenograft; HLA, human leukocyte antigen; GVHD, graft-versus-host disease; PK/PD, pharmacokinetics/pharmacodynamics; 2D, two-dimensional; 3D, three-dimensional.

**Table 3:** Representative immunosuppressive TME states, molecular nodes, decision questions, and mechanism-matched intervention strategies.

State Dimension	Representative Molecular Signals	Interpretive/Pharmacological Question	Potential Intervention Strategy
T-cell dysfunction	PD-1/PD-L1; exhaustion programs; co-inhibitory receptors [34,36]	Are effectors present and suppressible through a defined checkpoint?	PD-1/PD-L1 or other checkpoint blockade; context-specific costimulation
Treg-dominant suppression	FOXP3- and CTLA-4-associated programs; suppressive cytokines [37,56]	Is regulatory-cell activity a causal local constraint or only a correlate?	Selective Treg depletion/reprogramming; cytokine modulation with safety gating
Myeloid suppression	TAM/MDSC states; CSF1–CSF1R; ARG1; PI3Kγ-linked programs [44,46,47]	Does myeloid perturbation restore effector function without unacceptable systemic toxicity?	CSF1R/PI3Kγ/ARG1-directed strategies; macrophage reprogramming
CAF/ matrix exclusion	TGF-β–TGFBR–SMAD; CXCL12–CXCR4; ECM architecture [41,42]	Is immune exclusion driven by a druggable stromal mechanism in this tumor state?	TGF-β ligand/receptor modulation; CXCR4/FAK/stromal strategies; matrix remodeling
Vascular/ hypoxic barrier	HIF program; VEGF–VEGFR; perfusion markers [48,49]	Can vascular normalization improve trafficking and intratumoral exposure?	Anti-VEGF/VEGFR or normalization strategies; hypoxia-aware delivery/scheduling
Adenosine/ metabolic suppression	CD39/CD73; extracellular adenosine; A2A receptor; nutrient stress [51,52]	Is metabolic immune suppression targetable at achievable exposure?	CD73/A2AR or other metabolic-checkpoint inhibition; combination with ICI
Antigenicity/ presentation	Neoantigen burden; HLA and antigen-processing competence [13,37]	Is recognition failure upstream of checkpoint suppression?	Vaccines, epigenetic modulation, STING/innate activation, cell therapy
Spatial immune niche	Cell–cell adjacency; tertiary lymphoid structures; local target expression [10,83,84]	Which compartment and spatially co-localized mechanism is a candidate to be perturbed?	Spatially qualified combination selection and delivery strategy

Abbreviations: HLA, human leukocyte antigen; PD-1, programmed cell death protein 1; PD-L1, programmed death-ligand 1; Treg, regulatory T cell; FOXP3, forkhead box P3; CTLA-4, cytotoxic T-lymphocyte-associated protein 4; TAM, tumor-associated macrophage; MDSC, myeloid-derived suppressor cell; ARG1, arginase 1; CAF, cancer-associated fibroblast; CSF1, colony-stimulating factor 1; CSF1R, CSF1 receptor; PI3Kγ, phosphoinositide 3-kinase gamma; TGF-β, transforming growth factor beta; TGFBR, TGF-β receptor; SMAD, SMAD-family signal transducer; CXCL12, C-X-C motif chemokine ligand 12; CXCR4, C-X-C motif chemokine receptor 4; ECM, extracellular matrix; VEGF, vascular endothelial growth factor; VEGFR, VEGF receptor; HIF, hypoxia-inducible factor; CD39, cluster of differentiation 39; CD73, cluster of differentiation 73; A2AR, adenosine A2A receptor; A2A, adenosine receptor subtype A2A; ICI, immune checkpoint inhibitor; FAK, focal adhesion kinase; STING, stimulator of interferon genes.

**Table 4:** Documented computational contributions and their experimental boundaries.

Study and Target	Computational Contribution	Reported Experimental Support	Claim Boundary
Congreve et al. [117] A2A receptor	Structure-based development of 1,2,4-triazine antagonists	Receptor antagonism and medicinal-chemistry characterization	A molecular-discovery contribution; not a TME clinical result
Borodovsky et al. [118] AZD4635 and A2A receptor	Structure-derived candidate and receptor–ligand structural interpretation	Binding, signaling and preclinical immune-function studies	Preclinical pharmacology; no inference of clinical benefit from a design score
Watson et al. [112] PD-L1 protein binders	Generative design of protein binding interfaces	Experimental binder screening including PD-L1	Binding is distinct from checkpoint blockade and in vivo efficacy

Abbreviations: TME, tumor microenvironment; PD-L1, programmed death-ligand 1; A2A, adenosine receptor subtype A2A.

**Table 5:** CADD and pharmacology layers, appropriate uses, validity checks, and claim boundaries for TME-reprogramming drug design.

CADD/Pharmacology Layer	Scientific Question and Appropriate Use	Minimum Validity Checks/Boundary Conditions	Defensible Output—And What It Cannot Establish
Target/structure qualification	Define whether the nominated protein, complex, pocket, or interface is sufficiently specified for the intended modality	Correct isoform/complex Oligomeric state Relevant cofactors/PTMs Local structural confidence Alternative conformations Pocket/interface persistence	Structure or pocket hypothesis with provenance Does not establish druggability, binding, or efficacy
System-state/environment definition	Define the molecular state actually being screened or simulated	Protonation/tautomer states Waters and ions Membrane environment Cofactors and metal coordination Ligand ionization Receptor state	Reproducible molecular-system definition Does not represent the entire TME or patient physiology
Molecular docking/scoring	Evaluate whether a ligand can adopt a physically plausible pose Support compound prioritization	Redocking/cross-docking where relevant Receptor ensemble Water/metal treatment Leakage control Stereochemistry Steric/energetic plausibility	Pose/ranking hypothesis Screening enrichment Does not establish affinity, target engagement, or efficacy
Ligand-based QSAR/pharmacophore/generative design	Identify plausible chemotypes or property profiles within a defined chemical domain	Scaffold/time/project-aware splits Applicability domain Activity cliffs Assay consistency Synthetic feasibility Novelty and diversity	Compound/property prioritization within a supported domain Does not establish prospective potency outside that domain
Molecular dynamics/enhanced sampling	Examine conformational states, contacts, waters, and allosteric transitions compatible with the modeled system	Independent replicas/convergence checks Starting-pose sensitivity Force-field sensitivity Protonation sensitivity Timescale adequacy Justified collective variables	Conformational ensembles Mechanistic hypotheses Pose stability is not a binding-affinity measurement
Free-energy calculations	Estimate relative or absolute binding energetics for an appropriate chemical series	Stable binding mode Phase-space overlap Perturbation-network design Charge/protonation treatment Buried-water sampling Cycle closure or replicated uncertainty	Binding-energy estimate with uncertainty Does not establish cellular target engagement, exposure, or clinical benefit
QM/MM/reactive modeling	Determine whether electronic structure is essential to the proposed mechanism	QM-region definition Boundary treatment Reaction coordinate Electronic-state assumptions Adequate conformational sampling Sensitivity analysis	Reaction-mechanism or energetic hypothesis Not a routine substitute for MD, FEP, or organism-level pharmacology
ADMET/PK/PD/selectivity	Determine whether the candidate can reach and modulate the target at tolerable and selective exposure	Endpoint-specific validation Applicability domain Off-target profile Plausible/measured exposure Dose–response Target-engagement threshold Species/context differences	Developability and exposure constraints In silico ADMET does not replace measured PK, safety, or pharmacodynamics
Formulation/tissue delivery	Determine whether the modality can reach the relevant tumor compartment in active form	Stability Formulation Perfusion/interstitial transport Cellular uptake Spatial distribution Manufacturability Systemic vs. local exposure	Delivery and exposure hypothesis Systemic administration does not establish intratumoral delivery or response

Abbreviations: CADD, computer-aided drug design; TME, tumor microenvironment; PK/PD, pharmacokinetics/pharmacodynamics; PTM, post-translational modification; QM/MM, quantum mechanics/molecular mechanics; MD, molecular dynamics; FEP, free-energy perturbation; ADMET, absorption, distribution, metabolism, excretion, and toxicity; QSAR, quantitative structure–activity relationship.

**Table 6:** Representative TME state-to-target molecular-design logic and evidence requirements across CADD, pharmacology, and translation.

TME State/Molecular Target	Mechanistic Evidence	Molecular-Design Requirement	Target-Engagement/Exposure Requirement	Translational Limitation
Immune-excluded/TGF-β–TGFBR–SMAD	TGF-β activity linked to T-cell exclusion Resistance observed across human and model contexts [38,39,40,41]	For small-molecule TGFBR strategies: define receptor state, pocket, and protonation assumptions Apply docking, MD, or free-energy analysis only when appropriate to the ligand series For ligand traps/antibodies: evaluate interface and developability requirements separately	Demonstrate pathway suppression in the relevant stromal/immune compartment Confirm exposure, schedule, and safety Binding alone is insufficient	Strong context dependence Negative bintrafusp alfa phase III outcome shows that dual-pathway rationale alone does not establish biomarker-defined clinical utility [119]
CAF-mediated exclusion/CXCL12–CXCR4	CAF-derived CXCL12 can restrict T-cell access CAF states remain heterogeneous [42,43]	Use ligand- or structure-informed optimization for CXCR4-directed small molecules where appropriate Apply different modality and delivery logic for CAF-directed interventions Match the computational workflow to the target class	Demonstrate axis-specific pharmacodynamic effects Show restoration of immune access Confirm achievable exposure	CAF composition varies by tumor type Chemokine dependence is model- and context-specific
Myeloid-suppressive/CSF1R or PI3Kγ	CSF1R blockade can alter suppressive macrophage states PI3Kγ perturbation can support myeloid reprogramming [44,45,46,47]	Distinguish antibody and small-molecule optimization requirements For kinase-directed compounds: assess selectivity, physicochemical properties, conformational state, and compartment-relevant exposure	Demonstrate myeloid target engagement Confirm state change and dose–response Assess systemic safety	Macrophage plasticity Compensatory pathways Systemic immune effects may decouple target inhibition from durable tumor control
Vascular/VEGF–VEGFR barrier	VEGF-associated dysfunction can impair immune trafficking Can reduce intratumoral drug and immune-cell delivery [48,49,50]	Consider receptor-directed molecular design together with vascular-normalization window Incorporate dose, schedule, and combination context Do not use potency as the sole design endpoint	Measure exposure Assess vascular/perfusion pharmacodynamics Confirm effects on immune trafficking	Excessive pathway suppression may narrow the therapeutic window Poorly timed intervention may fail to improve delivery
Adenosine-rich suppression/CD73–adenosine–A2A receptor	Extracellular adenosine is a mechanistically supported immunosuppressive axis [51,52,53]	Apply different design criteria to A2A-receptor antagonists and CD73-directed biologics Evaluate structure, selectivity, and developability according to target class Avoid treating generic docking/FEP evidence as target-specific proof	Demonstrate receptor or enzymatic target engagement Confirm intratumoral pharmacodynamic effects Assess exposure and selectivity	Purinergic redundancy Compartment-specific adenosine production Systemic physiology may limit translation from biochemical potency

Abbreviations: TME, tumor microenvironment; CAF, cancer-associated fibroblast; CSF1R, CSF1 receptor; PI3Kγ, phosphoinositide 3-kinase gamma; TGF-β, transforming growth factor beta; TGFBR, TGF-β receptor; SMAD, SMAD-family signal transducer; CXCL12, C-X-C motif chemokine ligand 12; CXCR4, C-X-C motif chemokine receptor 4; VEGF, vascular endothelial growth factor; VEGFR, VEGF receptor; CD73, cluster of differentiation 73; A2A, adenosine receptor subtype A2A; MD, molecular dynamics; FEP, free-energy perturbation.

**Table 7:** Representative combination-therapy logics, evidence context, and key failure questions for escalation.

Combination Logic	Representative Example/Evidence	Evidence/Model Context	Key Failure Question
Checkpoint + stromal release	PD-1/PD-L1 blockade + TGF-β/CAF-axis intervention [38,39,41,119]	Human association Animal perturbation Phase III stress test	Is TGF-β/CAF-driven exclusion present in the selected population? Is incremental efficacy sufficient to justify added toxicity?
Checkpoint + vascular normalization	ICI + VEGF/VEGFR-directed normalization [48,49]	Mechanistic evidence Clinical-class evidence	Does the selected dose and schedule actually improve trafficking and intratumoral exposure?
Checkpoint + myeloid reprogramming	ICI + CSF1R/PI3Kγ-axis intervention [44,46,47]	Mechanistic evidence Preclinical perturbation	Does myeloid target engagement restore effector function without unacceptable systemic immune toxicity?
Checkpoint + metabolic relief	ICI + CD73/A2AR or related metabolic-checkpoint strategy [51,52]	Mechanistic evidence Emerging translational evidence	Is the suppressive metabolic axis active in the selected state? Can it be modulated at achievable intratumoral exposure?
Cell therapy + TME support	CAR-T/CAR-NK + trafficking, cytokine, stromal, or checkpoint support [166,167]	Clinical-trial landscape	Are antigen heterogeneity, trafficking, persistence, and local suppression addressed simultaneously?
Oncolytic/immune priming	Oncolytic strategy + checkpoint or conventional agent [167,168]	Preclinical evidence Clinical-class evidence	Is benefit schedule-dependent? Does the combination outperform each component with acceptable inflammation?
CADD/PK-constrained pairing	Docking/structure [19,20] MD/free energy [25,27] ADMET/PK [126,127] Delivery [121,138]	Foundational molecular-design and pharmacology evidence	Do both agents have compatible exposure, selectivity, formulation, and interaction profiles before synergy testing?

Abbreviations: CADD, computer-aided drug design; TME, tumor microenvironment; PD-1, programmed cell death protein 1; PD-L1, programmed death-ligand 1; CAF, cancer-associated fibroblast; CSF1R, CSF1 receptor; PI3Kγ, phosphoinositide 3-kinase gamma; TGF-β, transforming growth factor beta; VEGF, vascular endothelial growth factor; VEGFR, VEGF receptor; CD73, cluster of differentiation 73; A2AR, adenosine A2A receptor; ICI, immune checkpoint inhibitor; MD, molecular dynamics; ADMET, absorption, distribution, metabolism, excretion, and toxicity; PK, pharmacokinetics; CAR-T, chimeric antigen receptor T cell; CAR-NK, chimeric antigen receptor natural killer cell.

**Table 8:** Post-hoc IMPACT audit of INTR@PID Lung 037: diagnostic hypotheses, not established causes of trial failure.

IMPACT Responsibility	What the Phase III Result Establishes	Diagnostic Question Exposed by IMPACT	Prospective Design Implication
I—patient/model evidence	Randomized human comparison in PD-L1-high advanced NSCLC [119]	Did pre-escalation patient-derived evidence identify a subgroup that specifically required both TGF-β and PD-L1 modulation?	Link patient tissue, PDO, or explant perturbation to the intended biomarker-defined population
M—multi-omics mapping	PD-L1-based entry selection was clinically explicit	Did selection capture spatial TGF-β/CAF, myeloid, vascular, or metabolic states beyond PD-L1?	Predefine multimodal state variables corresponding to the added biological mechanism
P—TME phenotyping	Dual-target rationale was biologically plausible	Was a stable TGF-β-driven immune-excluded phenotype actually present in patients expected to benefit?	Require cross-assay and spatial phenotype qualification before mechanism-specific escalation
A—actionable AI/decision model	Trial evaluated treatment efficacy rather than an AI policy	Could a state-conditioned model have identified heterogeneity in benefit, futility, or toxicity with calibrated uncertainty?	Use AI for stratification and experiment prioritization, not as a substitute for randomized evaluation
C—combination/pharmacology	No superiority; Grade 3–4 treatment-related adverse events: 42.4% vs. 13.2% [119]	Was the incremental pathway coverage worth the exposure and toxicity cost in the selected population?	Jointly optimize mechanism, dose/schedule, PK/PD, toxicity, and biomarker enrichment
T—translational feedback	The trial did not establish superiority in the enrolled population [119]	What does the result imply for target-state definition and future combination claims?	Narrow, redesign, or discontinue the hypothesis rather than preserve the original claim unchanged

Abbreviations: IMPACT, in vivo and patient-derived systems, multi-omics mapping, phenotyping of TME states, actionable AI integration, combination-therapy prioritization, and translational testing; AI, artificial intelligence; TME, tumor microenvironment; PDO, patient-derived organoid; PK/PD, pharmacokinetics/pharmacodynamics; PD-L1, programmed death-ligand 1; CAF, cancer-associated fibroblast; TGF-β, transforming growth factor beta; NSCLC, non-small-cell lung cancer.

**Table 9:** Translational evidence gates, minimum support for escalation, HOLD/STOP signals, and corrective actions.

Gate	Minimum Evidence for Escalation	HOLD/STOP Signal	Decision/Corrective Action
G1. Data identity & QC	Sample/compound/structure traceability Donor-level independence Leakage audit	Identity mismatch Batch dominance Pseudoreplication Scaffold/target leakage	PASS: identity and independence are traceable HOLD: correctable QC or leakage problem STOP: identity cannot be resolved
G2. Molecular plausibility	Mechanism-linked target state Correct isoform/complex Adequate structure/pocket confidence	Target absent or inaccessible Uncertain pocket/complex State not reproducible	PASS: target, state, and modality are mutually plausible HOLD: refine state or structure STOP: unsupported target hypothesis
G3. Chemical evidence	Orthogonal pose checks Binding/biophysical support MD/FEP where scientifically justified	Unstable or physically invalid pose Failed binding Poor selectivity Nonconvergent energetics	PASS: chemically and biophysically coherent support HOLD: redesign or replicate STOP: failed binding or irreconcilable chemical evidence
G4. Target engagement/perturbation	Cellular target engagement Mechanism-linked rescue or rechallenge	Activity without target engagement No state rescue Off-target phenotype	PASS: engagement plus mechanism-consistent perturbation HOLD: revise mechanism or compound STOP: unsupported target-specific claim
G5. PK/PD & safety	Achievable exposure Dose–response Tissue penetration Toxicity/selectivity assessment	Exposure below effect threshold Incompatible schedule Unacceptable toxicity	PASS: achievable and tolerable exposure HOLD: optimize dose, formulation, or chemistry STOP: therapeutic window not credible
G6. Patient-derived transportability	Relevant PDO/explant/PDX or humanized-system evidence Explicit model caveats	Discordant response Effect restricted to unsupported context	PASS: transportability supported in relevant systems HOLD: narrow biomarker or model scope STOP: persistent discordance prevents broad extrapolation
G7. Prospective clinical utility	Predefined population Endpoint and biomarker Safety monitoring Prospective evaluation	No incremental utility Calibration failure Workflow or safety harm	PASS: predefined net clinical utility with acceptable safety HOLD: narrow indication or redesign evaluation STOP: clinical-utility claim unsupported

Abbreviations: PDO, patient-derived organoid; PDX, patient-derived xenograft; PK/PD, pharmacokinetics/pharmacodynamics; MD, molecular dynamics; FEP, free-energy perturbation; QC, quality control.

**Table 10:** Recommended evaluation domains matched to the scientific and pharmacological claim being made.

Domain	Representative Metrics/Checks	Why It Matters
Data/representation	Sample identity Batch-mixing metrics Missing-modality robustness Reproducibility	Prevents technical structure from being misinterpreted as biology
Prediction	AUROC/AUPRC Brier score Calibration error Decision-curve analysis	Separates discrimination from decision-relevant confidence
TME state	Cross-assay concordance State/cluster stability Pathway consistency	Tests whether inferred biological states are reproducible
CADD/molecular design	Structure/pocket confidence Redocking/cross-docking Scaffold/target-aware split Binding confirmation MD/FEP convergence Selectivity and ADMET checks	Prevents molecular scores from being interpreted as binding, target engagement, or efficacy
Counterfactual/perturbation	Effect-direction accuracy Treatment-response calibration Sensitivity to confounding assumptions	Prevents observational association from being presented as intervention evidence
Combination ranking	Top-k hit rate Synergy correlation Dose–surface error Toxicity-aware utility	Tests whether prioritized combinations are experimentally useful rather than merely highly ranked
External validity	Site/model/cancer-type shift Temporal shift OOD detection	Defines intended-use boundaries
Translational testing	PDO/PDX effect size Concordance across model classes Rescue/rechallenge	Links computational hypotheses to functional biology
Operational/governance	Provenance completeness Explainability Human override Auditability	Supports reproducible and accountable deployment

Abbreviations: CADD, computer-aided drug design; TME, tumor microenvironment; PDO, patient-derived organoid; PDX, patient-derived xenograft; MD, molecular dynamics; FEP, free-energy perturbation; ADMET, absorption, distribution, metabolism, excretion, and toxicity; AUROC, area under the receiver operating characteristic curve; AUPRC, area under the precision–recall curve; OOD, out of distribution.

## Data Availability

No new data were created or analyzed in this study. Data sharing is not applicable to this article.

## Supplementary Materials

The following supporting information can be downloaded at https://www.mdpi.com/article/10.3390/ijms27219766/s1. Extended review comparisons, representative evidence mapping, data and AI summaries, implementation records, search concepts, coding schema, and the reference-to-claim map are provided in the accompanying Supplementary Material.

## References

[1] Lipkova J., Chen R.J., Chen B., Lu M.Y., Barbieri M., Shao D., Vaidya A.J., Chen C., Zhuang L., Williamson D.F.K. (2022). Artificial intelligence for multimodal data integration in oncology. Cancer Cell.

[2] Acosta J.N., Falcone G.J., Rajpurkar P., Topol E.J. (2022). Multimodal biomedical AI. Nat. Med..

[3] Steyaert S., Pizurica M., Nagaraj D., Khandelwal P., Hernandez-Boussard T., Gentles A.J., Gevaert O. (2023). Multimodal data fusion for cancer biomarker discovery with deep learning. Nat. Mach. Intell..

[4] Zhuang L., Park S.H., Skates S.J., Prosper A.E., Aberle D.R., Hsu W. (2026). Advancing Precision Oncology Through Modeling of Longitudinal and Multimodal Data. IEEE Rev. Biomed. Eng..

[5] Li J., Zhang L., Yu Z., Bao Z., Li D., Wang L. (2026). The impact of AI on modern oncology from early detection to personalized cancer treatment. npj Precis. Oncol..

[6] Blanchard Z., Brown E.A., Ghazaryan A., Welm A.L. (2025). PDX models for functional precision oncology and discovery science. Nat. Rev. Cancer.

[7] Polak R., Zhang E.T., Kuo C.J. (2024). Cancer organoids 2.0: Modelling the complexity of the tumour immune microenvironment. Nat. Rev. Cancer.

[8] Pauli C., Hopkins B.D., Prandi D., Shaw R., Fedrizzi T., Sboner A., Sailer V., Augello M., Puca L., Rosati R. (2017). Personalized in vitro and in vivo cancer models to guide precision medicine. Cancer Discov..

[9] Gao H., Korn J.M., Ferretti S., Monahan J.E., Wang Y., Singh M., Zhang C., Schnell C., Yang G., Zhang Y. (2015). High-throughput screening using patient-derived tumor xenografts to predict clinical trial drug response. Nat. Med..

[10] Pelka K., Hofree M., Chen J.H., Sarkizova S., Pirl J.D., Jorgji V., Bejnood A., Dionne D., Ge W.H., Xu K.H. (2021). Spatially organized multicellular immune hubs in human colorectal cancer. Cell.

[11] Keren L., Bosse M., Marquez D., Angoshtari R., Jain S., Varma S., Yang S.R., Kurian A., Van Valen D., West R. (2018). A structured tumor-immune microenvironment in triple negative breast cancer revealed by multiplexed ion beam imaging. Cell.

[12] Bagaev A., Kotlov N., Nomie K., Svekolkin V., Gafurov A., Isaeva O., Osokin N., Kozlov I., Frenkel F., Gancharova O. (2021). Conserved pan-cancer microenvironment subtypes predict response to immunotherapy. Cancer Cell.

[13] Thorsson V., Gibbs D.L., Brown S.D., Wolf D., Bortone D.S., Ou Yang T.H., Porta-Pardo E., Gao G.F., Plaisier C.L., Eddy J.A. (2018). The immune landscape of cancer. Immunity.

[14] Abramson J., Adler J., Dunger J., Evans R., Green T., Pritzel A., Ronneberger O., Willmore L., Ballard A.J., Bambrick J. (2024). Accurate structure prediction of biomolecular interactions with AlphaFold 3. Nature.

[15] Jumper J., Evans R., Pritzel A., Green T., Figurnov M., Ronneberger O., Tunyasuvunakool K., Bates R., Žídek A., Potapenko A. (2021). Highly accurate protein structure prediction with AlphaFold. Nature.

[16] Kitchen D.B., Decornez H., Furr J.R., Bajorath J. (2004). Docking and scoring in virtual screening for drug discovery: Methods and applications. Nat. Rev. Drug Discov..

[17] Warren G.L., Andrews C.W., Capelli A.-M., Clarke B., LaLonde J., Lambert M.H., Lindvall M., Nevins N., Semus S.F., Senger S. (2006). A critical assessment of docking programs and scoring functions. J. Med. Chem..

[18] Su M., Yang Q., Du Y., Feng G., Liu Z., Li Y., Wang R. (2019). Comparative assessment of scoring functions: The CASF-2016 update. J. Chem. Inf. Model..

[19] Paggi J.M., Pandit A., Dror R.O. (2024). The art and science of molecular docking. Annu. Rev. Biochem..

[20] McNutt A.T., Francoeur P., Aggarwal R., Masuda T., Meli R., Ragoza M., Sunseri J., Koes D.R. (2021). GNINA 1.0: Molecular docking with deep learning. J. Cheminform..

[21] Corso G., Stärk H., Jing B., Barzilay R., Jaakkola T. DiffDock: Diffusion Steps, Twists, and Turns for Molecular Docking. Proceedings of the Eleventh International Conference on Learning Representations (ICLR 2023).

[22] Francoeur P.G., Masuda T., Sunseri J., Jia A., Iovanisci R.B., Snyder I., Koes D.R. (2020). Three-dimensional convolutional neural networks and a cross-docked data set for structure-based drug design. J. Chem. Inf. Model..

[23] Buttenschoen M., Morris G.M., Deane C.M. (2024). PoseBusters: AI-based docking methods fail to generate physically valid poses or generalise to novel sequences. Chem. Sci..

[24] Hollingsworth S.A., Dror R.O. (2018). Molecular dynamics simulation for all. Neuron.

[25] De Vivo M., Masetti M., Bottegoni G., Cavalli A. (2016). Role of molecular dynamics and related methods in drug discovery. J. Med. Chem..

[26] Bernardi R.C., Melo M.C.R., Schulten K. (2015). Enhanced sampling techniques in molecular dynamics simulations of biological systems. Biochim. Biophys. Acta (BBA)-Gen. Subj..

[27] Qian R., Xue J., Xu Y., Huang J. (2024). Alchemical Transformations and Beyond: Recent Advances and Real-World Applications of Free Energy Calculations in Drug Discovery. J. Chem. Inf. Model..

[28] Mey A.S.J.S., Allen B.K., Bruce Macdonald H.E., Chodera J.D., Hahn D.F., Kuhn M., Michel J., Mobley D.L., Naden L.N., Prasad S. (2020). Best practices for alchemical free energy calculations. Living J. Comput. Mol. Sci..

[29] Wang L., Wu Y., Deng Y., Kim B., Pierce L., Krilov G., Lupyan D., Robinson S., Dahlgren M.K., Greenwood J. (2015). Accurate and reliable prediction of relative ligand binding potency in prospective drug discovery by way of a modern free-energy calculation protocol and force field. J. Am. Chem. Soc..

[30] Schindler C.E.M., Baumann H., Blum A., Böse D., Buchstaller H.-P., Burgdorf L., Cappel D., Chekler E., Czodrowski P., Dorsch D. (2020). Large-scale assessment of binding free energy calculations in active drug discovery projects. J. Chem. Inf. Model..

[31] Raghavan B., Paulikat M., Ahmad K., Callea L., Rizzi A., Ippoliti E., Mandelli D., Bonati L., De Vivo M., Carloni P. (2023). Drug Design in the Exascale Era: A Perspective from Massively Parallel QM/MM Simulations. J. Chem. Inf. Model..

[32] Senn H.M., Thiel W. (2009). QM/MM methods for biomolecular systems. Angew. Chem. Int. Ed..

[33] van der Kamp M.W., Mulholland A.J. (2013). Combined quantum mechanics/molecular mechanics (QM/MM) methods in computational enzymology. Biochemistry.

[34] Pardoll D.M. (2012). The blockade of immune checkpoints in cancer immunotherapy. Nat. Rev. Cancer.

[35] Ribas A., Wolchok J.D. (2018). Cancer immunotherapy using checkpoint blockade. Science.

[36] Wei S.C., Duffy C.R., Allison J.P. (2018). Fundamental mechanisms of immune checkpoint blockade therapy. Cancer Discov..

[37] Chen D.S., Mellman I. (2017). Elements of cancer immunity and the cancer-immune set point. Nature.

[38] Mariathasan S., Turley S.J., Nickles D., Castiglioni A., Yuen K., Wang Y., Kadel E.E., Koeppen H., Astarita J.L., Cubas R. (2018). TGFβ attenuates tumour response to PD-L1 blockade by contributing to exclusion of T cells. Nature.

[39] Tauriello D.V.F., Palomo-Ponce S., Stork D., Berenguer-Llergo A., Badia-Ramentol J., Iglesias M., Sevillano M., Ibiza S., Cañellas A., Hernando-Momblona X. (2018). TGFβ drives immune evasion in genetically reconstituted colon cancer metastasis. Nature.

[40] Batlle E., Massagué J. (2019). Transforming growth factor-β signaling in immunity and cancer. Immunity.

[41] Derynck R., Turley S.J., Akhurst R.J. (2021). TGFβ biology in cancer progression and immunotherapy. Nat. Rev. Clin. Oncol..

[42] Feig C., Jones J.O., Kraman M., Wells R.J.B., Deonarine A., Chan D.S., Connell C.M., Roberts E.W., Zhao Q., Caballero O.L. (2013). Targeting CXCL12 from FAP-expressing carcinoma-associated fibroblasts synergizes with anti-PD-L1 immunotherapy in pancreatic cancer. Proc. Natl. Acad. Sci. USA.

[43] Elyada E., Bolisetty M., Laise P., Flynn W.F., Courtois E.T., Burkhart R.A., Teinor J.A., Belleau P., Biffi G., Lucito M.S. (2019). Cross-species single-cell analysis of pancreatic ductal adenocarcinoma reveals antigen-presenting cancer-associated fibroblasts. Cancer Discov..

[44] Ries C.H., Cannarile M.A., Hoves S., Benz J., Wartha K., Runza V., Rey-Giraud F., Pradel L.P., Feuerhake F., Klaman I. (2014). Targeting tumor-associated macrophages with anti-CSF-1R antibody reveals a strategy for cancer therapy. Cancer Cell.

[45] Pyonteck S.M., Akkari L., Schuhmacher A.J., Bowman R.L., Sevenich L., Quail D.F., Olson O.C., Quick M.L., Huse J.T., Teijeiro V. (2013). CSF-1R inhibition alters macrophage polarization and blocks glioma progression. Nat. Med..

[46] Kaneda M.M., Messer K.S., Ralainirina N., Li H., Leem C.J., Gorjestani S., Woo G., Nguyen A.V., Figueiredo C.C., Foubert P. (2016). PI3Kγ is a molecular switch that controls immune suppression. Nature.

[47] De Henau O., Rausch M., Winkler D., Campesato L.F., Liu C., Cymerman D.H., Budhu S., Ghosh A., Pink M., Tchaicha J. (2016). Overcoming resistance to checkpoint blockade therapy by targeting PI3Kγ in myeloid cells. Nature.

[48] Fukumura D., Kloepper J., Amoozgar Z., Duda D.G., Jain R.K. (2018). Enhancing cancer immunotherapy using antiangiogenics: Opportunities and challenges. Nat. Rev. Clin. Oncol..

[49] Jain R.K. (2013). Normalizing tumor microenvironment to treat cancer: Bench to bedside to biomarkers. J. Clin. Oncol..

[50] Voron T., Colussi O., Marcheteau E., Pernot S., Nizard M., Pointet A.L., Latreche S., Bergaya S., Benhamouda N., Tanchot C. (2015). VEGF-A modulates expression of inhibitory checkpoints on CD8+ T cells in tumors. J. Exp. Med..

[51] Leone R.D., Emens L.A. (2018). Targeting adenosine for cancer immunotherapy. J. Immunother. Cancer.

[52] Allard D., Turcotte M., Stagg J. (2017). Targeting A2 adenosine receptors in cancer. Immunol. Cell Biol..

[53] Cekic C., Linden J. (2016). Purinergic regulation of the immune system. Nat. Rev. Immunol..

[54] Joyce J.A., Fearon D.T. (2015). T cell exclusion, immune privilege, and the tumor microenvironment. Science.

[55] Quail D.F., Joyce J.A. (2013). Microenvironmental regulation of tumor progression and metastasis. Nat. Med..

[56] Binnewies M., Roberts E.W., Kersten K., Chan V., Fearon D.F., Merad M., Coussens L.M., Gabrilovich D.I., Ostrand-Rosenberg S., Hedrick C.C. (2018). Understanding the tumor immune microenvironment (TIME) for effective therapy. Nat. Med..

[57] Byrne A.T., Alférez D.G., Amant F., Annibali D., Arribas J., Biankin A.V., Bruna A., Budinská E., Caldas C., Chang D.K. (2017). Interrogating open issues in cancer precision medicine with patient-derived xenografts. Nat. Rev. Cancer.

[58] Dijkstra K.K., Cattaneo C.M., Weeber F., Chalabi M., van de Haar J., Fanchi L.F., Slagter M., van der Velden D.L., Kaing S., Kelderman S. (2018). Generation of tumor-reactive T cells by co-culture of peripheral blood lymphocytes and tumor organoids. Cell.

[59] Cherkasov A., Muratov E.N., Fourches D., Varnek A., Baskin I.I., Cronin M., Dearden J., Gramatica P., Martin Y.C., Todeschini R. (2014). QSAR modeling: Where have you been? Where are you going to?. J. Med. Chem..

[60] Wu Z., Ramsundar B., Feinberg E.N., Gomes J., Geniesse C., Pappu A.S., Leswing K., Pande V. (2018). MoleculeNet: A benchmark for molecular machine learning. Chem. Sci..

[61] Mysinger M.M., Carchia M., Irwin J.J., Shoichet B.K. (2012). Directory of Useful Decoys, Enhanced (DUD-E): Better ligands and decoys for better benchmarking. J. Med. Chem..

[62] Sheridan R.P. (2013). Time-split cross-validation as a method for estimating the goodness of prospective prediction. J. Chem. Inf. Model..

[63] Mobley D.L., Gilson M.K. (2017). Predicting binding free energies: Frontiers and benchmarks. Annu. Rev. Biophys..

[64] Han D., Lu J., Fan B., Lu W., Xue Y., Wang M., Liu T., Cui S., Gao Q., Duan Y. (2024). Lysine-Specific Demethylase 1 Inhibitors: A Comprehensive Review Utilizing Computer-Aided Drug Design Technologies. Molecules.

[65] Tian W., Hu Y., Gao X., Yang J., Jiang W. (2025). Computer-Aided Drug Design Across Breast Cancer Subtypes: Methods, Applications and Translational Outlook. Int. J. Mol. Sci..

[66] Hidalgo M., Amant F., Biankin A.V., Budinská E., Byrne A.T., Caldas C., Clarke R.B., de Jong S., Jonkers J., Mælandsmo G.M. (2014). Patient-Derived Xenograft Models: An Emerging Platform for Translational Cancer Research. Cancer Discov..

[67] Neal J.T., Li X., Zhu J., Giangarra V., Grzeskowiak C.L., Ju J., Liu I.H., Chiou S.-H., Salahudeen A.A., Smith A.R. (2018). Organoid Modeling of the Tumor Immune Microenvironment. Cell.

[68] Voabil P., de Bruijn M., Roelofsen L.M., Hendriks S.H., Brokamp S., van den Braber M., Broeks A., Sanders J., Herzig P., Zippelius A. (2021). An ex vivo tumor fragment platform to dissect response to PD-1 blockade in cancer. Nat. Med..

[69] Bruna A., Rueda O.M., Greenwood W., Batra A.S., Callari M., Batra R.N., Pogrebniak K., Sandoval J., Cassidy J.W., Tufegdzic-Vidakovic A. (2016). A biobank of breast cancer explants with preserved intra-tumor heterogeneity to screen anticancer compounds. Cell.

[70] Drost J., Clevers H. (2018). Organoids in cancer research. Nat. Rev. Cancer.

[71] Tiriac H., Belleau P., Engle D.D., Plenker D., Deschênes A., Somerville T.D.D., Froeling F.E.M., Burkhart R.A., Denroche R.E., Jang G.H. (2018). Organoid profiling identifies common responders to chemotherapy in pancreatic cancer. Cancer Discov..

[72] Kopper O., de Witte C.J., Lõhmussaar K., Valle-Inclan J.E., Hami N., Kester L., Balgobind A.V., Korving J., Proost N., Begthel H. (2019). An organoid platform for ovarian cancer captures intra- and interpatient heterogeneity. Nat. Med..

[73] Jung J., Kim J., Lim H.K., Kim K.M., Lee Y.S., Park J.S., Yoon D.S. (2017). Establishing a colorectal cancer liver metastasis patient-derived tumor xenograft model for the evaluation of personalized chemotherapy. Ann. Surg. Treat. Res..

[74] Ooft S.N., Weeber F., Dijkstra K.K., McLean C.M., Kaing S., van Werkhoven E., Schipper L., Hoes L., Vis D.J., van de Haar J. (2019). Patient-derived organoids can predict response to chemotherapy in metastatic colorectal cancer patients. Sci. Transl. Med..

[75] Vlachogiannis G., Hedayat S., Vatsiou A., Jamin Y., Fernández-Mateos J., Khan K., Lampis A., Eason K., Huntingford I., Burke R. (2018). Patient-derived organoids model treatment response of metastatic gastrointestinal cancers. Science.

[76] Esposito A., Agostini A., Quero G., Piro G., Priori L., Caggiano A., Scaglione G., Battaglia A., Calegari M.A., Salvatore L. (2024). Colorectal cancer patients-derived immunity-organoid platform unveils cancer-specific tissue markers associated with immunotherapy resistance. Cell Death Dis..

[77] Biswas N., Chakrabarti S. (2020). Artificial intelligence (AI)-based systems biology approaches in multi-omics data analysis of cancer. Front. Oncol..

[78] Heo Y.J., Hwa C., Lee G.H., Park J.M., An J.Y. (2021). Integrative multi-omics approaches in cancer research: From biological networks to clinical subtypes. Mol. Cells.

[79] Raufaste-Cazavieille V., Santiago R., Droit A. (2022). Multi-omics analysis: Paving the path toward achieving precision medicine in cancer treatment and immuno-oncology. Front. Mol. Biosci..

[80] Liu M., Villazon J., Forjaz A., Tian X., Fan R., Wang S., Shi L., Wirtz D., Kiemen A.L. (2026). 3D multi-omics tumour atlases: From technology to biology and clinical translation. Nat. Rev. Cancer.

[81] Wu S.Z., Al-Eryani G., Roden D.L., Junankar S., Harvey K., Andersson A., Thennavan A., Wang C., Torpy J.R., Bartonicek N. (2021). A single-cell and spatially resolved atlas of human breast cancers. Nat. Genet..

[82] Jackson H.W., Fischer J.R., Zanotelli V.R.T., Ali H.R., Mechera R., Soysal S.D., Moch H., Muenst S., Varga Z., Weber W.P. (2020). The single-cell pathology landscape of breast cancer. Nature.

[83] Helmink B.A., Reddy S.M., Gao J., Zhang S., Basar R., Thakur R., Yizhak K., Sade-Feldman M., Blando J., Han G. (2020). B cells and tertiary lymphoid structures promote immunotherapy response. Nature.

[84] Cabrita R., Lauss M., Sanna A., Donia M., Skaarup Larsen M., Mitra S., Johansson I., Phung B., Harbst K., Vallon-Christersson J. (2020). Tertiary lymphoid structures improve immunotherapy and survival in melanoma. Nature.

[85] Williams H.L., Frei A.L., Koessler T., Berger M.D., Dawson H., Michielin O., Zlobec I. (2024). The current landscape of spatial biomarkers for prediction of response to immune checkpoint inhibition. npj Precis. Oncol..

[86] Lan Z., Yang Y., Li L., Wang C., Sun Z., Wang Q., Liu Y. (2025). Spatial omics technology potentially promotes the progress of tumor immunotherapy. Br. J. Cancer.

[87] Addala V., Newell F., Pearson J.V., Redwood A., Robinson B.W., Creaney J., Waddell N. (2024). Computational immunogenomic approaches to predict response to cancer immunotherapies. Nat. Rev. Clin. Oncol..

[88] Greenwald N.F., Nederlof I., Sowers C., Ding D.Y., Park S., Kong A., Houlahan K.E., Varra S.R., de Graaf M., Geurts V. (2026). Temporal and spatial composition of the tumor microenvironment predicts response to immune checkpoint inhibition in metastatic TNBC. Nat. Cancer.

[89] Chen J.H., Nieman L.T., Spurrell M., Jorgji V., Elmelech L., Richieri P., Xu K.H., Madhu R., Parikh M., Zamora I. (2024). Human lung cancer harbors spatially organized stem-immunity hubs associated with response to immunotherapy. Nat. Immunol..

[90] Argelaguet R., Velten B., Arnol D., Dietrich S., Zenz T., Marioni J.C., Buettner F., Huber W., Stegle O. (2018). Multi-Omics Factor Analysis—A framework for unsupervised integration of multi-omics data sets. Mol. Syst. Biol..

[91] Cui H., Wang C., Maan H., Pang K., Luo F., Duan N., Wang B. (2024). scGPT: Toward building a foundation model for single-cell multi-omics using generative AI. Nat. Methods.

[92] Hao M., Gong J., Zeng X., Liu C., Guo Y., Cheng X., Wang T., Ma J., Zhang X., Song L. (2024). Large-scale foundation model on single-cell transcriptomics. Nat. Methods.

[93] Aliazis K., Christofides A., Shah R., Yeo Y.Y., Jiang S., Charest A., Boussiotis V.A. (2025). The tumor microenvironment’s role in the response to immune checkpoint blockade. Nat. Cancer.

[94] Lambrechts D., Wauters E., Boeckx B., Aibar S., Nittner D., Burton O., Bassez A., Decaluwé H., Pircher A., Van den Eynde K. (2018). Phenotype molding of stromal cells in the lung tumor microenvironment. Nat. Med..

[95] Kieffer Y., Hocine H.R., Gentric G., Pelon F., Bernard C., Bourachot B., Lameiras S., Albergante L., Bonneau C., Guyard A. (2020). Single-cell analysis reveals fibroblast clusters linked to immunotherapy resistance in cancer. Cancer Discov..

[96] Jerby-Arnon L., Shah P., Cuoco M.S., Rodman C., Su M.-J., Melms J.C., Leeson R., Kanodia A., Mei S., Lin J.-R. (2018). A cancer cell program promotes T cell exclusion and resistance to checkpoint blockade. Cell.

[97] Sade-Feldman M., Yizhak K., Bjorgaard S.L., Ray J.P., de Boer C.G., Jenkins R.W., Lieb D.J., Chen J.H., Frederick D.T., Barzily-Rokni M. (2018). Defining T cell states associated with response to checkpoint immunotherapy in melanoma. Cell.

[98] Xie X., Yu T., Li X., Zhang N., Foster L.J., Peng C., Huang W., He G. (2023). Recent advances in targeting the “undruggable” proteins: From drug discovery to clinical trials. Signal Transduct. Target. Ther..

[99] Hincapié-López M., Marín-Alfonso J., Romero-Riaño E., Núñez-Rodríguez R.A., Pabón-Martínez Y.V. (2025). Antisense Versus Antigene in the Computer-Aided Design of Triplex-Forming Oligonucleotides (TFO): Insights from a Dual-Method Review, Combining Bibliometric and Systematic Review. Int. J. Mol. Sci..

[100] Wagner J., Carvajal A.I., Bracher A., Beck F., Wan W., Bohn S., Körner R., Baumeister W., Fernandez-Busnadiego R., Hartl F.U. (2024). Visualizing chaperonin function in situ by cryo-electron tomography. Nature.

[101] Trott O., Olson A.J. (2010). AutoDock Vina: Improving the speed and accuracy of docking with a new scoring function, efficient optimization, and multithreading. J. Comput. Chem..

[102] Friesner R.A., Banks J.L., Murphy R.B., Halgren T.A., Klicic J.J., Mainz D.T., Repasky M.P., Knoll E.H., Shelley M., Perry J.K. (2004). Glide: A new approach for rapid, accurate docking and scoring. 1. Method and assessment of docking accuracy. J. Med. Chem..

[103] Halgren T.A., Murphy R.B., Friesner R.A., Beard H.S., Frye L.L., Pollard W.T., Banks J.L. (2004). Glide: A new approach for rapid, accurate docking and scoring. 2. Enrichment factors in database screening. J. Med. Chem..

[104] Irwin J.J., Shoichet B.K. (2005). ZINC—A free database of commercially available compounds for virtual screening. J. Chem. Inf. Model..

[105] Morris G.M., Huey R., Lindstrom W., Sanner M.F., Belew R.K., Goodsell D.S., Olson A.J. (2009). AutoDock4 and AutoDockTools4: Automated docking with selective receptor flexibility. J. Comput. Chem..

[106] Stärk H., Ganea O.-E., Pattanaik L., Barzilay R., Jaakkola T. EquiBind: Geometric Deep Learning for Drug Binding Structure Prediction. Proceedings of the 39th International Conference on Machine Learning (ICML 2022).

[107] Lu W., Wu Q., Zhang J., Rao J., Li C., Zheng S. TANKBind: Trigonometry-Aware Neural Networks for Drug-Protein Binding Structure Prediction. Proceedings of the 36th Conference on Neural Information Processing Systems (NeurIPS 2022).

[108] Yang K., Swanson K., Jin W., Coley C., Eiden P., Gao H., Guzman-Perez A., Hopper T., Kelley B., Mathea M. (2019). Analyzing learned molecular representations for property prediction. J. Chem. Inf. Model..

[109] Mayr A., Klambauer G., Unterthiner T., Steijaert M., Wegner J.K., Ceulemans H., Clevert D.A., Hochreiter S. (2018). Large-scale comparison of machine learning methods for drug target prediction on ChEMBL. Chem. Sci..

[110] Schneuing A., Harris C., Du Y., Didi K., Jamasb A., Igashov I., Du W., Gomes C., Blundell T.L., Lio P. (2024). Structure-based drug design with equivariant diffusion models. Nat. Comput. Sci..

[111] Hu Q., Sun C., He H., Xu J., Liu D., Zhang W., Shi S., Zhang K., Li H. (2025). Target-aware 3D molecular generation based on guided equivariant diffusion. Nat. Commun..

[112] Watson J.L., Juergens D., Bennett N.R., Trippe B.L., Yim J., Eisenach H.E., Ahern W., Borst A.J., Ragotte R.J., Milles L.F. (2023). De novo design of protein structure and function with RFdiffusion. Nature.

[113] Brown N., Fiscato M., Segler M.H.S., Vaucher A.C. (2019). GuacaMol: Benchmarking models for de novo molecular design. J. Chem. Inf. Model..

[114] Polykovskiy D., Zhebrak A., Sanchez-Lengeling B., Golovanov S., Tatanov O., Belyaev S., Kurbanov R., Artamonov A., Aladinskiy V., Veselov M. (2020). Molecular Sets (MOSES): A benchmarking platform for molecular generation models. Front. Pharmacol..

[115] Olivecrona M., Blaschke T., Engkvist O., Chen H. (2017). Molecular de-novo design through deep reinforcement learning. J. Cheminform..

[116] Walters W.P., Murcko M. (2020). Assessing the impact of generative AI on medicinal chemistry. Nat. Biotechnol..

[117] Congreve M., Andrews S.P., Doré A.S., Hollenstein K., Hurrell E., Langmead C.J., Mason J.S., Ng I.W., Tehan B., Zhukov A. (2012). Discovery of 1,2,4-triazine derivatives as adenosine A2A antagonists using structure based drug design. J. Med. Chem..

[118] Borodovsky A., Barbon C.M., Wang Y., Ye M., Prickett L., Chandra D., Shaw J., Deng N., Sachsenmeier K., Clarke J.D. (2020). Small molecule AZD4635 inhibitor of A2AR signaling rescues immune cell function including CD103+ dendritic cells enhancing anti-tumor immunity. J. Immunother. Cancer.

[119] Cho B.C., Lee J.S., Wu Y.-L., Cicin I., Dols M.C., Ahn M.-J., Cuppens K., Veillon R., Nadal E., Dias J.M. (2023). Bintrafusp Alfa Versus Pembrolizumab in Patients with Treatment-Naive, Programmed Death-Ligand 1-High Advanced NSCLC: A Randomized, Open-Label, Phase 3 Trial. J. Thorac. Oncol..

[120] Chen B., Liu S., Xia H., Li X., Zhang Y. (2025). Computer-Aided Drug Design in Research on Chinese Materia Medica: Methods, Applications, Advantages, and Challenges. Pharmaceutics.

[121] Dewhirst M.W., Secomb T.W. (2017). Transport of drugs from blood vessels to tumour tissue. Nat. Rev. Cancer.

[122] Cournia Z., Allen B., Sherman W. (2017). Relative binding free energy calculations in drug discovery: Recent advances and practical considerations. J. Chem. Inf. Model..

[123] Aldeghi M., Heifetz A., Bodkin M.J., Knapp S., Biggin P.C. (2016). Accurate calculation of the absolute free energy of binding for drug molecules. Chem. Sci..

[124] Ross G.A., Russell E., Deng Y., Lu C., Harder E.D., Abel R., Wang L. (2020). Enhancing water sampling in free energy calculations with grand canonical Monte Carlo. J. Chem. Theory Comput..

[125] Brunk E., Rothlisberger U. (2015). Mixed quantum mechanical/molecular mechanical molecular dynamics simulations of biological systems in ground and electronically excited states. Chem. Rev..

[126] Pires D.E.V., Blundell T.L., Ascher D.B. (2015). pkCSM: Predicting small-molecule pharmacokinetic and toxicity properties using graph-based signatures. J. Med. Chem..

[127] Xiong G., Wu Z., Yi J., Fu L., Yang Z., Hsieh C., Yin M., Zeng X., Wu C., Lu A. (2021). ADMETlab 2.0: An integrated online platform for accurate and comprehensive predictions of ADMET properties. Nucleic Acids Res..

[128] Lipinski C.A., Lombardo F., Dominy B.W., Feeney P.J. (2001). Experimental and computational approaches to estimate solubility and permeability in drug discovery and development settings. Adv. Drug Deliv. Rev..

[129] Daina A., Michielin O., Zoete V. (2017). SwissADME: A free web tool to evaluate pharmacokinetics, drug-likeness and medicinal chemistry friendliness of small molecules. Sci. Rep..

[130] Fu L., Shi S., Yi J., Wang N., He Y., Wu Z., Peng J., Deng Y., Wang W., Wu C. (2024). ADMETlab 3.0: An updated comprehensive online ADMET prediction platform enhanced with broader coverage, improved performance, API functionality and decision support. Nucleic Acids Res..

[131] Banerjee P., Eckert A.O., Schrey A.K., Preissner R. (2018). ProTox-II: A webserver for the prediction of toxicity of chemicals. Nucleic Acids Res..

[132] Cumming J.G., Davis A.M., Muresan S., Haeberlein M., Chen H. (2013). Chemical predictive modelling to improve compound quality. Nat. Rev. Drug Discov..

[133] Waring M.J., Arrowsmith J., Leach A.R., Leeson P.D., Mandrell S., Owen R.M., Pairaudeau G., Pennie W.D., Pickett S.D., Wang J. (2015). An analysis of the attrition of drug candidates from four major pharmaceutical companies. Nat. Rev. Drug Discov..

[134] Cook D., Brown D., Alexander R., March R., Morgan P., Satterthwaite G., Pangalos M.N. (2014). Lessons learned from the fate of AstraZeneca’s drug pipeline: A five-dimensional framework. Nat. Rev. Drug Discov..

[135] Morgan P., Van Der Graaf P.H., Arrowsmith J., Feltner D.E., Drummond K.S., Wegner C.D., Street S.D.A. (2012). Can the flow of medicines be improved? Fundamental pharmacokinetic and pharmacological principles toward improving Phase II survival. Drug Discov. Today.

[136] Wilhelm S., Tavares A.J., Dai Q., Ohta S., Audet J., Dvorak H.F., Chan W.C.W. (2016). Analysis of nanoparticle delivery to tumours. Nat. Rev. Mater..

[137] Rosenblum D., Joshi N., Tao W., Karp J.M., Peer D. (2018). Progress and challenges towards targeted delivery of cancer therapeutics. Nat. Commun..

[138] Sindhwani S., Syed A.M., Ngai J., Kingston B.R., Maiorino L., Rothschild J., MacMillan P., Zhang Y., Rajesh N.U., Hoang T. (2020). The entry of nanoparticles into solid tumours. Nat. Mater..

[139] Mitchell M.J., Billingsley M.M., Haley R.M., Wechsler M.E., Peppas N.A., Langer R. (2021). Engineering precision nanoparticles for drug delivery. Nat. Rev. Drug Discov..

[140] Liu Z., Ji P., Liu H., Yu L., Zhang S.-M., Liu P., Zhang X.-Z., Luo G.-F., Shang Z. (2023). FNIII14 peptide-enriched membrane nanocarrier to disrupt stromal barriers through reversing CAFs for augmenting drug penetration in tumors. Nano Lett..

[141] Lu X., Jin J., Wu Y., Lin J., Zhang X., Lu S., Zhang J., Zhang C., Ren M., Chen H. (2025). Self-assembled PROTACs enable protein degradation to reprogram the tumor microenvironment for synergistically enhanced colorectal cancer immunotherapy. Bioact. Mater..

[142] Petzold G., Fischer E.S., Thomä N.H. (2016). Structural basis of lenalidomide-induced CK1α degradation by the CRL4CRBN ubiquitin ligase. Nature.

[143] Liu F., Beck S., Yang L., Luo H., Zhang K. (2026). Advancing AI for multi-omics and clinical data integration in basic and translational cancer research. Nat. Rev. Cancer.

[144] Kline A., Wang H., Li Y., Dennis S., Hutch M., Xu Z., Wang F., Cheng F., Luo Y. (2022). Multimodal machine learning in precision health: A scoping review. npj Digit. Med..

[145] Stahlschmidt S.R., Ulfenborg B., Synnergren J. (2022). Multimodal deep learning for biomedical data fusion: A review. Brief. Bioinform..

[146] Jiménez-Luna J., Grisoni F., Schneider G. (2020). Drug discovery with explainable artificial intelligence. Nat. Mach. Intell..

[147] Vamathevan J., Clark D., Czodrowski P., Dunham I., Ferran E., Lee G., Li B., Madabhushi A., Shah P., Spitzer M. (2019). Applications of machine learning in drug discovery and development. Nat. Rev. Drug Discov..

[148] Truhn D., Eckardt J.N., Ferber D., Kather J.N. (2024). Large language models and multimodal foundation models for precision oncology. npj Precis. Oncol..

[149] Niu C., Wang G. (2026). The current and future landscape of AI foundation models for cancer management. Nat. Commun..

[150] Hao Y., Qiu Z., Holmes J., Löckenhoff C.E., Liu W., Ghassemi M., Kalantari S. (2025). Large language model integrations in cancer decision-making: A systematic review and meta-analysis. npj Digit. Med..

[151] Sutanto H., Fetarayani D. (2025). Integrating artificial intelligence into small molecule development for precision cancer immunomodulation therapy. npj Drug Discov..

[152] Schneider P., Walters W.P., Plowright A.T., Sieroka N., Listgarten J., Goodnow R.A., Fisher J., Jansen J.M., Duca J.S., Rush T.S. (2020). Rethinking drug design in the artificial intelligence era. Nat. Rev. Drug Discov..

[153] Hernán M.A., Robins J.M. (2020). Causal Inference: What If.

[154] Pearl J., Bareinboim E. (2014). External Validity: From Do-Calculus to Transportability Across Populations. Stat. Sci..

[155] Murphy S.A. (2003). Optimal Dynamic Treatment Regimes. J. R. Stat. Soc. Ser. B Stat. Methodol..

[156] Laber E.B., Lizotte D.J., Qian M., Pelham W.E., Murphy S.A. (2014). Dynamic treatment regimes: Technical challenges and applications. Electron. J. Stat..

[157] Williams N.T., Hoffman K.L., Díaz I., Rudolph K.E. (2024). Learning optimal dynamic treatment regimes from longitudinal data. Am. J. Epidemiol..

[158] Zhao Y., Wang J., Kim J., Rodriguez F., Linos E., Na R., Al-Jamal K.T., Li X. (2026). Machine learning and deep learning-based drug-drug interactions prediction: A systematic review focused on anticancer drugs. npj Precis. Oncol..

[159] Yadav B., Wennerberg K., Aittokallio T., Tang J. (2015). Searching for drug synergy in complex dose-response landscapes using an interaction potency model. Comput. Struct. Biotechnol. J..

[160] Ianevski A., Giri A.K., Aittokallio T. (2020). SynergyFinder 2.0: Visual analytics of multi-drug combination synergies. Nucleic Acids Res..

[161] Bliss C.I. (1939). The toxicity of poisons applied jointly. Ann. Appl. Biol..

[162] Berenbaum M.C. (1989). What is synergy?. Pharmacol. Rev..

[163] Menden M.P., Wang D., Mason M.J., Szalai B., Bulusu K.C., Guan Y., Yu T., Kang J., Jeon M., Wolfinger R. (2019). Community assessment to advance computational prediction of cancer drug combinations in a pharmacogenomic screen. Nat. Commun..

[164] Li L., Zhang H., Zheng C., Su Y. (2025). A review of deep learning approaches for drug synergy prediction in cancer. npj Drug Discov..

[165] Holbeck S.L., Camalier R., Crowell J.A., Govindharajulu J.P., Hollingshead M., Anderson L.W., Polley E., Rubinstein L., Srivastava A., Wilsker D. (2017). The National Cancer Institute ALMANAC: A comprehensive screening resource for the detection of anticancer drug pairs with enhanced therapeutic activity. Cancer Res..

[166] Peng L., Sferruzza G., Yang L., Zhou L., Chen S. (2024). CAR-T and CAR-NK as cellular cancer immunotherapy for solid tumors. Cell. Mol. Immunol..

[167] Galon J., Bruni D. (2019). Approaches to treat immune hot, altered and cold tumours with combination immunotherapies. Nat. Rev. Drug Discov..

[168] Shalhout S.Z., Miller D.M., Emerick K.S., Kaufman H.L. (2023). Therapy with oncolytic viruses: Progress and challenges. Nat. Rev. Clin. Oncol..

