# Supplementary material for: Computer-Aided Drug Design for Immunosuppressive Tumor Microenvironment Reprogramming: A Critical Review
Source: Int J Mol Sci. 2026 Oct 30;27(21):9766. doi: 10.3390/ijms27219766 (PMC13763026; doi:10.3390/ijms27219766)
Supplement: Supplementary file 1 [file ijms-27-09766-s001.zip › ijms-4581207-supplementary.pdf]

Supplementary Materials

Computer-Aided Drug Design for Immunosuppressive Tumor Microenvironment Reprogramming: A Critical Review

These supplementary materials provide extended evidence maps, search concepts, and methodological records for the critical review. Reference numbers correspond to the main manuscript bibliography. Terms used below include tumor microenvironment (TME), computer-aided drug design (CADD), and artificial intelligence (AI). IMPACT denotes in vivo and patient-derived systems, multi-omics mapping, phenotyping of TME states, actionable AI integration, combination-therapy prioritization, and translational testing. Capability ratings and evidence classifications are interpretive syntheses with the limitations described in the main text.

S1 Extended comparisons and evidence maps

Table S1. Positioning against representative neighboring reviews and review-oriented syntheses.

Review focus	Representative reviews	Typical boundary	Added perspective here
Multimodal biomedical AI	Multimodal biomedical and precision-health reviews [2,144]	Broad modalities and clinical tasks; often prediction-centric	Intervention-oriented TME reprogramming loop
Multimodal oncology and precision oncology	Oncology integration and foundation-model perspectives [1,5,148]	Cancer-specific integration; variable emphasis on causal action	Patient-derived state/action evidence with explicit evidence gates
Longitudinal multimodal precision oncology	Longitudinal and multimodal oncology review [4]	Dynamic biomarkers and longitudinal modeling	Explicit separation of one-step response, transportability, and policy value
Patient-derived models	PDX and functional precision-oncology reviews [6,57]	Strong model-system emphasis; limited AI decision architecture	Question-specific model capabilities linked to AI and intervention claims
Tumor organoids and TME models	Organoid/TME reviews [7,70]	Human-relevant ex-vivo modeling; limited closed-loop decision layer	AI ranking, uncertainty, and model-to-human qualification
Spatial and immunogenomic TME	TME checkpoint-response review [93]; spatial reviews [85,86]; immunogenomics review [87]	Biomarkers and tissue architecture	TME state treated as a testable intervention object
Cancer foundation models and decision support	Cancer foundation-model editorials and reviews [148–150]	Generalist representation and reasoning	Foundation models positioned as components under provenance and abstention requirements
Drug-combination AI	Synergy prediction [164]; drug–drug interaction prediction [158]	Efficacy ranking and interaction/toxicity risks address different questions	Joint consideration of efficacy, safety and patient-derived validation
CADD, molecular modeling, and AI-assisted drug design	Docking/scoring [16,18,19]; AI/ML docking [20,21,23]; MD/free energy [25,27,28]; ADMET/AI medicinal chemistry [127,147,151]	Often molecule- or benchmark-centric; patient/TME context and transportability may fall outside scope	TME state → targetability → molecular design → pharmacology → patient-derived validation

Abbreviations: CADD, computer-aided drug design; AI, artificial intelligence; TME, tumor microenvironment; PDX, patient-derived xenograft; MD, molecular dynamics; ADMET, absorption, distribution, metabolism, excretion, and toxicity; ML, machine learning.

Table S2. Externally anchored representative evidence map showing evidence role, inferential relationship, model system, validation maturity, and principal limitation.

Study	Evidence role	Context / model system	Task or intervention	Evidence relationship	Validation maturity	Principal limitation
Neal [67]	Direct solid-tumor translational	Multiple solid tumors / air-liquid-interface PDO	Checkpoint perturbation	Perturbational	Orthogonal/external	Ex-vivo culture duration and selection limit long-term inference
Voabil [68]	Direct solid-tumor translational	Human tumor fragments	Programmed cell death protein 1 (PD-1) blockade response	Perturbational	Orthogonal/external	Ex-vivo response does not equal randomized patient benefit
Mariathasan [38]	Direct solid-tumor translational	Urothelial cancer cohort + mouse models	Transforming growth factor beta (TGF-β) / programmed death-ligand 1 (PD-L1) immune exclusion	Observational + perturbational	Orthogonal/external	Cross-system transport requires explicit qualification
Tauriello [39]	Direct solid-tumor translational	Colorectal cancer metastasis / mouse model	TGF-β-driven immune evasion	Perturbational	Orthogonal/external	Genetically defined model may not span human heterogeneity
Vlachogiannis [75]	Direct solid-tumor translational	Metastatic gastrointestinal cancer / PDO	Patient-specific drug response	Perturbational	Orthogonal/external	Culture context incompletely represents systemic immunity
Dijkstra [58]	Direct solid-tumor translational	Tumor organoid + autologous lymphocytes	Tumor-reactive T-cell generation	Perturbational	Orthogonal/external	Autologous co-culture still lacks full systemic context
Jerby-Arnon [96]	Direct solid-tumor translational	Melanoma / human single-cell data + mouse models	T-cell exclusion and checkpoint resistance program	Observational + perturbational	Orthogonal/external	Clinical association and mouse intervention do not establish patient treatment benefit
Pelka [10]	Direct solid-tumor translational	Human colorectal cancer / spatial single-cell profiling	Spatial multicellular immune hubs	Observational/ predictive	Orthogonal/external	Spatial association does not identify intervention effect
Lambrechts [94]	Direct solid-tumor translational	Human lung cancer / single-cell profiling	Stromal-state remodeling	Observational/ predictive	Orthogonal/external	Cross-sectional state may not be longitudinally stable
Kieffer [95]	Direct solid-tumor translational	Breast-cancer CAF profiling + functional co-culture	CAF states linked to immunotherapy resistance	Observational + perturbational	Orthogonal/external	CAF–Treg functional results and response associations have distinct experimental scopes
Jung [73]	Direct solid-tumor translational	Colorectal liver metastasis / PDX	Single-donor PDX chemotherapy testing	Perturbational	Internal	Single donor and immunodeficient host limit generalization
Abramson [14]	Foundational/transferable methodology	Biomolecular complexes / structure prediction	Protein–ligand and biomolecular complex structure prediction	N/A	Orthogonal/external	Predicted complex geometry does not establish affinity, target engagement, or efficacy
McNutt [20]	Foundational/transferable methodology	Protein–ligand docking / benchmark	Deep-learning-assisted molecular docking and scoring	N/A	Orthogonal/external	Benchmark docking accuracy depends on pocket definition and does not establish biological response
Acosta [2]	Review/synthesis	Multimodal biomedical AI	Cross-modal integration principles	N/A	N/A	Broad biomedical scope; not TME-intervention evidence
Zhuang [4]	Review/synthesis	Longitudinal multimodal precision oncology	Dynamic disease and response modeling	N/A	N/A	Review-level evidence; does not validate a specific intervention
Corso [21]	Foundational/transferable methodology	Protein–ligand docking / PDBBind	Diffusion-based blind docking	N/A	Orthogonal/external	Pose success on structural benchmarks does not establish affinity or target-context validity
Cho [119]	Direct solid-tumor translational	PD-L1-high advanced NSCLC / randomized phase III	Bintrafusp alfa (TGF-β trap + PD-L1 blockade) vs. pembrolizumab	Prospective interventional	Prospective/co-clinical	No superiority over pembrolizumab; higher grade 3–4 treatment-related adverse events
Buttenschoen [23]	Foundational/transferable methodology	Protein–ligand docking / novel-sequence benchmark	Physical validity and generalization audit for AI docking	N/A	Orthogonal/external	RMSD success can coexist with physically invalid poses; benchmark scope still differs from therapeutic efficacy
Greenwald [88]	Direct solid-tumor translational	Metastatic triple-negative breast cancer / longitudinal human trial cohort	Spatial and temporal TME features associated with immune-checkpoint inhibitor response	Observational/ predictive	Internal/orthogonal	One trial cohort; on-treatment prediction is not pretreatment clinical utility
Chen [89]	Direct solid-tumor translational	Non-small-cell lung cancer / human spatial profiling	Stem-immunity hubs associated with programmed cell death protein 1 blockade response	Observational/ predictive	Internal	Spatial association requires independent prospective validation before clinical use
Esposito [76]	Direct solid-tumor translational	Colorectal cancer / autologous patient-derived immuno-organoid + human specimens	Immune-checkpoint inhibitor resistance markers and perturbational rescue	Observational + perturbational	Orthogonal/external	Ex-vivo immune-organoid response does not establish prospective patient benefit
Qian [27]	Review/synthesis	Drug-discovery free-energy calculations	Relative/absolute binding free-energy applications	N/A	N/A	Accuracy and convergence depend on system preparation, sampling, and perturbation design

Abbreviations: AI, artificial intelligence; TME, tumor microenvironment; N/A, not applicable; PDO, patient-derived organoid; PDX, patient-derived xenograft; PD-1, programmed cell death protein 1; PD-L1, programmed death-ligand 1; CAF, cancer-associated fibroblast; TGF-β, transforming growth factor beta; NSCLC, non-small-cell lung cancer; Treg, regulatory T cell; RMSD, root-mean-square deviation.

Table S3. Multi-omics modalities, TME information, computational representations, and common failure modes.

Modality	TME information	AI representation	Common failure mode
WES/WGS	Mutations, copy-number alterations, neoantigen substrate	Variant/pathway embeddings	Tumor purity and low-frequency clone loss
Bulk RNA-seq	Pathways; immune/stromal mixtures	Gene-set or transformer embeddings	Cell-composition confounding
scRNA-seq / scATAC-seq	Cell states, transitions, regulatory programs	Cell embeddings, latent states, graphs	Batch effects, dissociation bias, sparse counts
Spatial transcriptomics/ proteomics	Niches, exclusion, cell–cell adjacency	Spatial graphs / patch embeddings	Resolution differences and registration error
Proteomics/ phosphoproteomics	Functional signaling and pathway activity	Protein/pathway embeddings	Platform coverage and dynamic range
Digital pathology / imaging	Morphology, architecture, spatial phenotype	CNN, Vision Transformer, or whole-slide-image representations	Site/scanner shift and annotation bias
Treatment + clinical metadata	Dose, timing, toxicity, outcomes	Structured temporal context	Treatment-selection bias and missingness

Abbreviations: AI, artificial intelligence; TME, tumor microenvironment; WES, whole-exome sequencing; WGS, whole-genome sequencing; RNA, ribonucleic acid; scRNA-seq, single-cell RNA sequencing; scATAC-seq, single-cell assay for transposase-accessible chromatin sequencing; CNN, convolutional neural network.

Table S4. AI model families and their best-supported roles in TME-oriented precision oncology.

Model family	Strength	Risk / limitation	Best-fit role
Gradient boosting / elastic models	Strong small-sample baseline; Relatively transparent feature contribution	Limited representation of complex cross-modal interactions	Benchmarking; Tabular and pathway-level integration
CNN / Vision Transformer (ViT)	Strong image and pathology representation	Scanner/site shift; Annotation burden; Potential tile-level pseudoreplication	Digital pathology; Imaging-derived phenotyping
Graph neural network (GNN)	Explicit representation of molecular, spatial, or cellular relationships	Graph-construction assumptions can dominate results; Edge definitions may encode uncertain biology	Cell–cell networks; Drug–target/pathway networks; Spatial TME modeling
Multimodal transformer	Flexible cross-modal interaction; Cross-attention across heterogeneous context	Data-intensive; Missing-modality sensitivity; Calibration challenges	Intermediate multimodal fusion
Single-cell foundation model	Transfer across cell types and tasks; Reusable cellular representations	Pretraining-domain mismatch; Cancer-specific calibration may be weak	Cell-state embedding; Perturbation-transfer hypotheses
LLM / multimodal foundation model	Evidence organization; Retrieval and tool orchestration; Heterogeneous reasoning interface	Hallucination; Provenance errors; Weak mechanistic validation	Evidence synthesis and orchestration under expert oversight
Bayesian / ensemble uncertainty quantification	Decision-aware uncertainty estimation; Can support abstention and experiment prioritization	Computational overhead; Uncertainty can remain unreliable under OOD shift	Uncertainty gating; Next-experiment prioritization

Abbreviations: TME, tumor microenvironment; OOD, out of distribution; CNN, convolutional neural network; GNN, graph neural network; LLM, large language model.

Table S5. Multi-model implementation blueprint linking biological assets, CADD, pharmacology, and translational evidence hand-offs.

Layer	Core artifacts	Example methods	Output to next layer
Model systems	Patient tissue; PDO; Explant; PDX; Humanized-system metadata	Identity/QC; Lineage and passage; Histology; Species-aware processing; Treatment metadata	Biologically scoped multi-model sample set
Data asset	WES; RNA-seq; scRNA-seq; Spatial data; Proteomics; Pathology; Exposure data	QC; Harmonization; Annotation; Provenance tracking; Missingness modeling	Traceable multimodal feature store
TME state / target	Cell composition; Pathways; Spatial niches; Receptor/ligand expression; Target isoform/complex	Deconvolution; Clustering; Spatial statistics; Graph/pathway analysis; Perturbational evidence	Mechanistic state and target hypothesis with uncertainty
CADD / chemistry	Structures; Pockets/interfaces; Compounds; Stereochemistry; Analog series	Structure qualification; Docking/virtual screening; Ligand-based models; MD/FEP; QM/MM when justified	Small, traceable candidate set with binding and chemistry hypotheses
Pharmacology	Binding; Target engagement; Selectivity; ADMET; PK/PD; Formulation	Biophysical assays; Cellular assays; Exposure modeling; Safety profiling	Achievable dose/schedule and mechanism-linked perturbation plan
Combination engine	TME state; Candidate actions; Toxicity constraints; Exposure constraints	Dose-surface experiments; Bayesian optimization; Pareto ranking	Mechanism-matched candidate combinations
Validation	Ex vivo/PDO/PDX evidence; Co-clinical data; Prospective outcomes	Rescue/rechallenge; Longitudinal analysis; Model triangulation	New evidence, failure signals, and model/compound updates
Governance	Data provenance; Structure provenance; Compound provenance; Model provenance; Human review	Versioning; Assay lineage; Model/compound cards; Audit trail	Bounded and reproducible intended use

Abbreviations: CADD, computer-aided drug design; TME, tumor microenvironment; PDO, patient-derived organoid; PDX, patient-derived xenograft; PK/PD, pharmacokinetics/pharmacodynamics; QM/MM, quantum mechanics/molecular mechanics; MD, molecular dynamics; FEP, free-energy perturbation; ADMET, absorption, distribution, metabolism, excretion, and toxicity; QC, quality control; WES, whole-exome sequencing; RNA, ribonucleic acid; scRNA-seq, single-cell RNA sequencing.

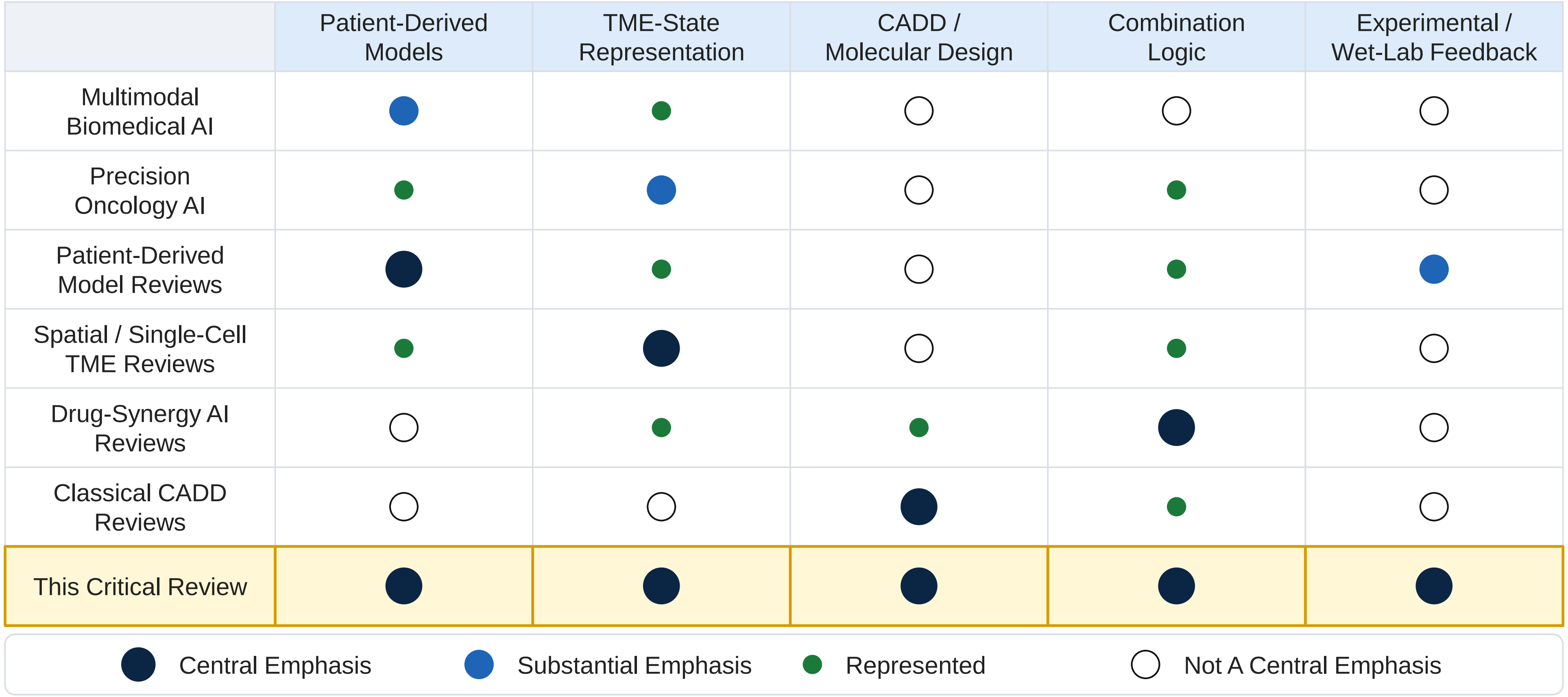

Figure S1. Contemporary review landscape. Marker size indicates qualitative, author-coded central emphasis across patient-derived models, TME-state representation, CADD/molecular design, combination logic, and experimental feedback. Open circles indicate that a dimension is not a central emphasis in the corresponding review family. marker sizes are not numerical scores or validated rankings. No arrows are used because the figure compares thematic coverage rather than direction, workflow, or causation. CADD, computer-aided drug design; TME, tumor microenvironment; AI, artificial intelligence.

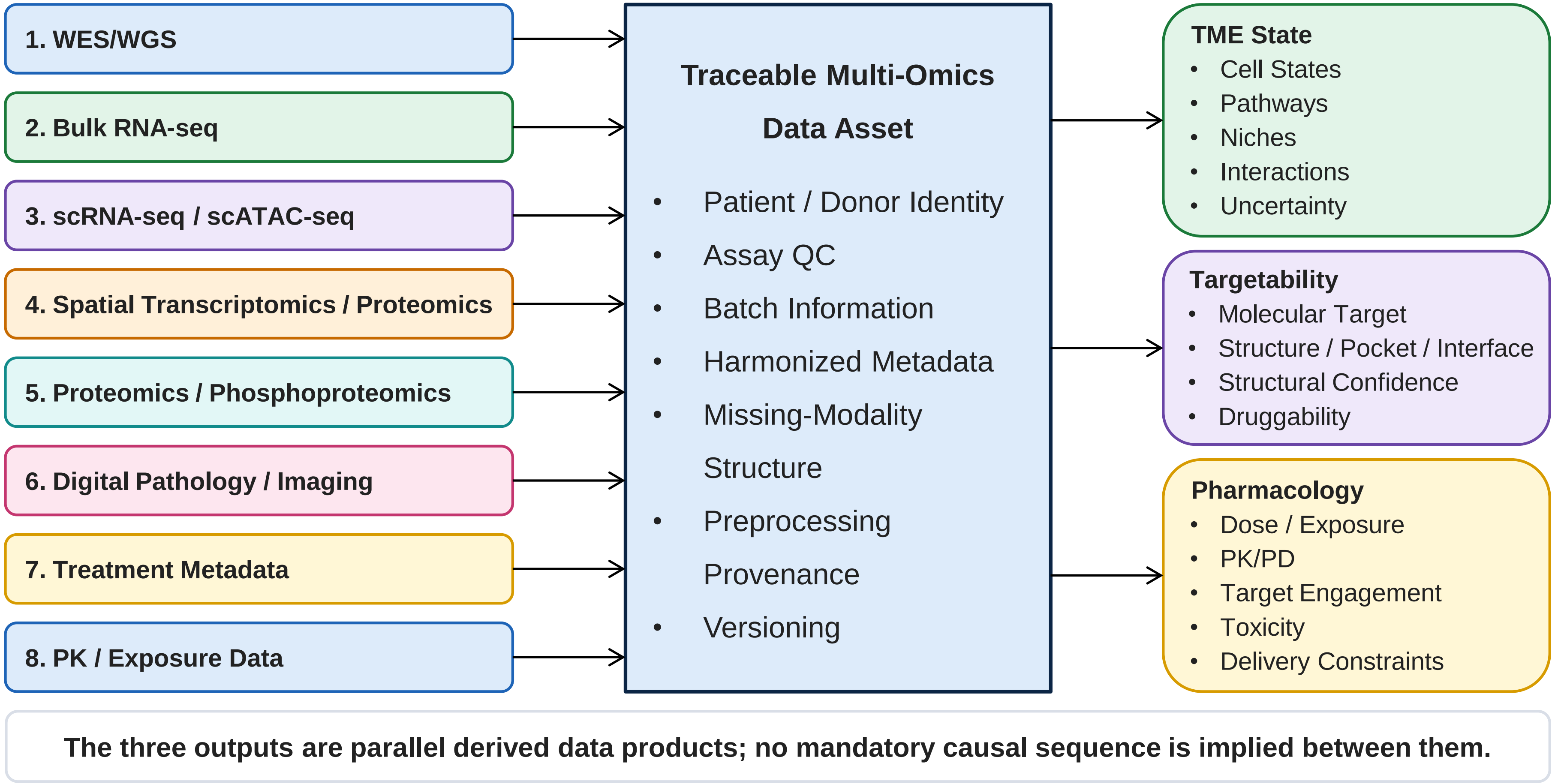

Figure S2. Multi-omics mapping as a traceable state-to-targetability data asset. Molecular assays, spatial context, pathology, treatment, and pharmacokinetic information are harmonized with identity, quality control, missingness, and provenance. TME state, molecular targetability, and pharmacology are represented as parallel auditable outputs; the figure does not imply a causal sequence among them. Abbreviations: WES/WGS, whole-exome/whole-genome sequencing; RNA-seq, ribonucleic acid sequencing; scRNA-seq, single-cell RNA sequencing; scATAC-seq, single-cell assay for transposase-accessible chromatin sequencing; TME, tumor microenvironment; QC, quality control; PK, pharmacokinetics; PK/PD, pharmacokinetics/ pharmacodynamics.

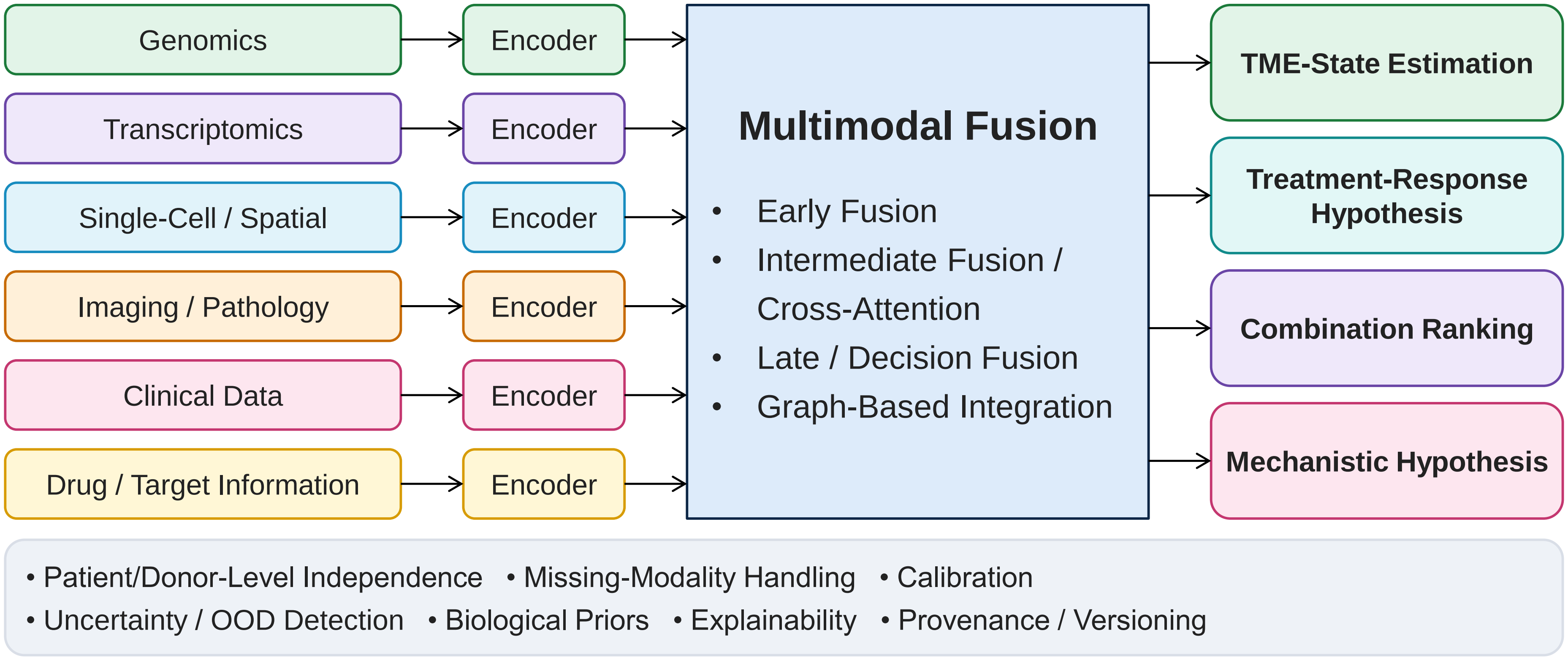

Figure S3. Artificial intelligence (AI) integration stack. Modality-specific encoders and fusion strategies generate state, response, or ranking hypotheses. Donor-level independence, missing-modality handling, calibration, uncertainty and out-of-distribution assessment, biological priors, explanation, and provenance operate as validation and governance constraints rather than as causal inputs. Abbreviations: TME, tumor microenvironment; OOD, out-of-distribution.

S2 Targeted literature identification

We identified literature through 15 October 2026 using PubMed, publisher records, and citation chaining. The search addressed TME biology, patient-derived models, molecular design, simulation, pharmacology, and AI-supported interpretation. Targeted concepts included native-state structural methods, three-dimensional generative design, stromal delivery, and protein degradation. The expressions below summarize these concepts; they are representative terms rather than verbatim database queries.

1. Protein-binder generation against programmed death-ligand 1 (PD-L1); RFdiffusion; pocket-conditioned ligand generation; DiffSBDD.
2. Cryo-electron tomography; in situ conformational states; spatial proteomics in tumors.
3. Adenosine A2A receptor antagonist discovery; structure-based design; AZD4635.
4. Fibroblast activation protein (FAP)-responsive delivery; cancer-associated fibroblasts (CAFs); stromal penetration; fibronectin-derived FNIII14 peptide.
5. Proteolysis-targeting chimeras (PROTACs); signal transducer and activator of transcription 3 (STAT3); β-catenin; molecular glues; casein kinase 1 alpha (CK1α); cereblon (CRBN).

Table S6 groups the broader search concepts used to identify relevant literature. Sources were selected for identifiable targets or methods, stated model systems, and interpretable validation. Selection was purposive and qualitative, without a completed exhaustive screening dataset or independent duplicate-coding assessment.

Table S6. Search-concept families used in the targeted critical review.

Concept family	Representative concepts	Purpose
TME biology	Tumor microenvironment; Immune suppression CAF; TAM; Treg; MDSC; Immune exclusion	Define reprogrammable TME states
Patient-derived models	PDX; Humanized PDX; Patient-derived organoid; Tumor explant; Co-clinical systems	Map model capabilities and validation levels
Multi-omics	WES; RNA-seq; scRNA-seq; Spatial transcriptomics; Proteomics; Multimodal oncology	Define data and integration layers
AI integration	Multimodal machine learning; Fusion; GNN; Transformer; Foundation model; Uncertainty	Map computational architectures
Response / combination	Treatment response; Predictive biomarker; Drug synergy; Combination therapy; Immunotherapy resistance	Connect state to intervention
Translation	External validation; Prospective validation; Clinical deployment; Digital twin; Governance	Define evidence gates and limitations
CADD / molecular informatics	Computer-aided drug design; Structure-/ligand-based design; Docking/scoring<br>Virtual screening; QSAR; Generative design; MD/FEP/QM/MM ADMET/PK/PD; Formulation/delivery	Connect TME target hypotheses to molecular design, developability, exposure, and falsifiable pharmacology

Abbreviations: CADD, computer-aided drug design; AI, artificial intelligence; TME, tumor microenvironment; PDX, patient-derived xenograft; PK/PD, pharmacokinetics/pharmacodynamics; Treg, regulatory T cell; TAM, tumor-associated macrophage; MDSC, myeloid-derived suppressor cell; CAF, cancer-associated fibroblast; QM/MM, quantum mechanics/molecular mechanics; MD, molecular dynamics; FEP, free-energy perturbation; ADMET, absorption, distribution, metabolism, excretion, and toxicity; QSAR, quantitative structure–activity relationship; WES, whole-exome sequencing; RNA, ribonucleic acid; scRNA-seq, single-cell RNA sequencing; GNN, graph neural network.

S3 Coding definitions and a template for further work

Table S7 provides a record schema for extending the evidence map. Table S2 presents the claim-specific interpretive synthesis used in this review. High (H), moderate (M), and low (L) capability labels refer to standard model classes under the rubric in main-text Section 2.2. They are qualitative assignments, with case-specific reassessment needed for engineered or borderline implementations.

Table S7. Suggested fields for a future systematic extension.

Block	Variables
Bibliographic	Author; Year; Venue; DOI; Peer-review status
Cancer context	Tumor type; Stage; Prior therapy; Sample source
Model system	Cell line; Spheroid; PDO; Explant; PDX; Humanized PDX; Clinical
Data modalities	Genomics; Transcriptomics; Single-cell; Spatial; Proteomics; Imaging; Clinical
TME phenotype	Immune/stromal populations; Spatial niche; Pathway; Dynamic state
AI	Encoder; Fusion; Foundation model; Graph model; UQ; XAI
Intervention	Drug(s); Immunotherapy; Cell therapy; Cytokine; Oncolytic virus; Dose/schedule
Causal support	Observational; Randomized; Perturbational; Proxy/instrumental; Longitudinal
Validation	Internal; External; Wet-lab; PDX; Prospective/co-clinical
Outcomes	Response; Synergy; Toxicity; Calibration; Mechanism; Clinical utility
Governance	Code/data availability; Model version; Provenance; Privacy; Human oversight
Evidence relationship	Observational/predictive; Perturbational; Prospective interventional
Validation maturity	Internal; Orthogonal/external; Prospective/co-clinical
Independence unit	Patient/donor; PDX lineage; Organoid line; Specimen; Mouse; Well; Cell; Tile
Transportability	Source model; Target population; Assumed invariant mechanisms; Known model differences
Author overlap / transparency	Author overlap: yes/no; Recorded for transparency only; does not alter evidence grade
Molecular target	Gene/protein; Isoform; Complex; Cell type; Subcellular/spatial compartment; Target-state evidence
Structure / CADD evidence	Experimental/predicted structure ID; Pocket/interface; Docking/scoring; Ligand-based model; MD/FEP/QM/MM; Benchmark split; Uncertainty
Pharmacology / developability	Binding; Target engagement; Selectivity; ADMET; PK/PD; Dose/schedule; Formulation/delivery; Toxicity

Abbreviations: CADD, computer-aided drug design; AI, artificial intelligence; TME, tumor microenvironment; PDO, patient-derived organoid; PDX, patient-derived xenograft; PK/PD, pharmacokinetics/pharmaco - dynamics; QM/MM, quantum mechanics/molecular mechanics; MD, molecular dynamics; FEP, free-energy perturbation; ADMET, absorption, distribution, metabolism, excretion, and toxicity; ID, identifier; UQ, uncertainty quantification; XAI, explainable artificial intelligence; DOI, digital object identifier.

S4 Reference to claim map

The following entries describe the roles assigned to references in this review. Methodological papers support method-level claims, not automatically TME efficacy. This map is an interpretive audit aid and is not an independently validated coding study.

Each of the 168 references is linked below to its specific analytical role and evidence type. Mixed studies are labeled with both observational and experimental components when relevant. These labels describe the source and its use in this review; they are not quality scores. Reference numbers are shared with the main manuscript and follow first appearance there.

[1] Lipkova, J. — Analytical role: Multimodal oncology integration and clinical applications. Evidence type: Review.

[2] Acosta, J.N. — Analytical role: Multimodal biomedical AI, fusion strategies and validation challenges. Evidence type: Review.

[3] Steyaert, S. — Analytical role: Deep-learning fusion for cancer biomarker discovery. Evidence type: Review.

[4] Zhuang, L. — Analytical role: Longitudinal multimodal modeling in precision oncology. Evidence type: Review.

[5] Li, J. — Analytical role: AI across cancer detection, profiling and drug discovery. Evidence type: Review.

[6] Blanchard, Z. — Analytical role: PDX models in functional precision oncology and discovery. Evidence type: Review.

[7] Polak, R. — Analytical role: Immune-TME representation and limitations of cancer organoids. Evidence type: Review.

[8] Pauli, C. — Analytical role: Paired patient-derived organoid and xenograft drug testing. Evidence type: Patient-derived experiments.

[9] Gao, H. — Analytical role: Large PDX panels for treatment-response screening. Evidence type: Preclinical experiments.

[10] Pelka, K. — Analytical role: Spatial multicellular immune hubs in colorectal cancer. Evidence type: Human observational.

[11] Keren, L. — Analytical role: Multiplexed ion-beam imaging of triple-negative breast cancer. Evidence type: Human observational.

[12] Bagaev, A. — Analytical role: Pan-cancer TME subtypes and immunotherapy-response associations. Evidence type: Human observational.

[13] Thorsson, V. — Analytical role: Pan-cancer immune classification and molecular correlates. Evidence type: Human observational/resource.

[14] Abramson, J. — Analytical role: AlphaFold 3 biomolecular-complex structure prediction. Evidence type: Computational method.

[15] Jumper, J. — Analytical role: AlphaFold protein-structure prediction and structural benchmarks. Evidence type: Computational method.

[16] Kitchen, D.B. — Analytical role: Molecular docking, scoring and virtual-screening methods. Evidence type: Review.

[17] Warren, G.L. — Analytical role: Comparative docking and scoring accuracy; limits of affinity ranking. Evidence type: Benchmark.

[18] Su, M. — Analytical role: CASF-2016 scoring, ranking, docking and screening assessment. Evidence type: Benchmark.

[19] Paggi, J.M. — Analytical role: Docking methodology, practical applications and limitations. Evidence type: Review.

[20] McNutt, A.T. — Analytical role: GNINA neural-network-assisted docking and rescoring. Evidence type: Method/tool.

[21] Corso, G. — Analytical role: DiffDock diffusion-based pose prediction for supplied ligands. Evidence type: Computational method.

[22] Francoeur, P.G. — Analytical role: CrossDocked data and convolutional scoring; evaluation and generalization. Evidence type: Method/benchmark.

[23] Buttenschoen, M. — Analytical role: Physical validity and novel-sequence generalization of docking poses. Evidence type: Benchmark.

[24] Hollingsworth, S.A. — Analytical role: Molecular dynamics principles and interpretation. Evidence type: Review.

[25] De Vivo, M. — Analytical role: Molecular dynamics and related methods in drug discovery. Evidence type: Review.

[26] Bernardi, R.C. — Analytical role: Enhanced conformational sampling in biomolecular simulation. Evidence type: Review.

[27] Qian, R. — Analytical role: Alchemical free-energy methods and drug-discovery applications. Evidence type: Review.

[28] Mey, A.S.J.S. — Analytical role: Setup, convergence, uncertainty and reporting of alchemical calculations. Evidence type: Methods guidance.

[29] Wang, L. — Analytical role: Relative-affinity calculations with prospective drug-discovery applications. Evidence type: Prospective discovery.

[30] Schindler, C.E.M. — Analytical role: Large-scale binding free-energy assessment in active discovery projects. Evidence type: Prospective discovery/benchmark.

[31] Raghavan, B. — Analytical role: Massively parallel QM/MM and illustrative drug-design applications. Evidence type: Perspective/method cases.

[32] Senn, H.M. — Analytical role: QM/MM treatment of biomolecular electronic and reaction processes. Evidence type: Review.

[33] van der Kamp, M.W. — Analytical role: QM/MM methods in computational enzymology. Evidence type: Review.

[34] Pardoll, D.M. — Analytical role: Immune-checkpoint blockade mechanisms and therapeutic rationale. Evidence type: Review.

[35] Ribas, A. — Analytical role: Cancer immunotherapy with checkpoint blockade. Evidence type: Review.

[36] Wei, S.C. — Analytical role: Mechanisms underlying immune-checkpoint blockade. Evidence type: Review.

[37] Chen, D.S. — Analytical role: Cancer-immunity set point and context-dependent response. Evidence type: Review.

[38] Mariathasan, S. — Analytical role: TGF-β-associated exclusion and combined TGF-β/PD-L1 blockade. Evidence type: Human association + mouse perturbation.

[39] Tauriello, D.V.F. — Analytical role: TGF-β-mediated immune evasion in colorectal-cancer metastasis models. Evidence type: Mouse perturbation.

[40] Batlle, E. — Analytical role: TGF-β signaling in immunity and cancer. Evidence type: Review.

[41] Derynck, R. — Analytical role: Context-dependent TGF-β biology and cancer immunotherapy. Evidence type: Review.

[42] Feig, C. — Analytical role: CAF-derived CXCL12/CXCR4 interference and anti-PD-L1 response in pancreatic cancer. Evidence type: Mouse perturbation.

[43] Elyada, E. — Analytical role: Antigen-presenting CAFs and fibroblast heterogeneity in pancreatic cancer. Evidence type: Human/mouse profiling + functional assays.

[44] Ries, C.H. — Analytical role: Anti-CSF1R macrophage targeting, including responses in diffuse-type giant-cell tumors. Evidence type: Preclinical + early clinical.

[45] Pyonteck, S.M. — Analytical role: CSF1R inhibition, macrophage polarization and glioma progression. Evidence type: Mouse perturbation.

[46] Kaneda, M.M. — Analytical role: Myeloid PI3Kγ signaling and immune suppression. Evidence type: Preclinical mechanism.

[47] De Henau, O. — Analytical role: PI3Kγ inhibition and checkpoint-blockade resistance. Evidence type: Mouse perturbation.

[48] Fukumura, D. — Analytical role: Antiangiogenic therapy, vascular normalization and immunotherapy combinations. Evidence type: Perspective/review.

[49] Jain, R.K. — Analytical role: Vascular/TME normalization, perfusion and delivery implications. Evidence type: Review/translational synthesis.

[50] Voron, T. — Analytical role: VEGF-A induction of inhibitory checkpoints on tumor CD8+ T cells. Evidence type: Mechanistic experiments.

[51] Leone, R.D. — Analytical role: Adenosine signaling as a cancer-immunotherapy target. Evidence type: Review.

[52] Allard, D. — Analytical role: A2 adenosine receptors in cancer. Evidence type: Review.

[53] Cekic, C. — Analytical role: Purinergic signaling in immune regulation. Evidence type: Review.

[54] Joyce, J.A. — Analytical role: T-cell exclusion and immune privilege in the TME. Evidence type: Review.

[55] Quail, D.F. — Analytical role: Microenvironmental regulation of tumor progression and metastasis. Evidence type: Review.

[56] Binnewies, M. — Analytical role: Tumor immune-microenvironment composition and therapeutic implications. Evidence type: Review.

[57] Byrne, A.T. — Analytical role: PDX model fidelity, passage effects and translational limitations. Evidence type: Review/opinion.

[58] Dijkstra, K.K. — Analytical role: Autologous lymphocyte–organoid co-culture and tumor-reactive T-cell generation. Evidence type: Patient-derived perturbation.

[59] Cherkasov, A. — Analytical role: QSAR applicability domains, validation and methodological practice. Evidence type: Review.
[60] Wu, Z. — Analytical role: MoleculeNet molecular-learning datasets and evaluation tasks. Evidence type: Benchmark/resource.
[61] Mysinger, M.M. — Analytical role: DUD-E ligand and property-matched decoy construction for screening evaluation. Evidence type: Benchmark resource.
[62] Sheridan, R.P. — Analytical role: Time-split versus random validation for prospective molecular prediction. Evidence type: Validation method.
[63] Mobley, D.L. — Analytical role: Binding free-energy prediction and benchmarking limitations. Evidence type: Review.
[64] Han, D. — Analytical role: CADD approaches to LSD1 inhibitor discovery. Evidence type: Review.
[65] Tian, W. — Analytical role: CADD applications across breast-cancer subtypes. Evidence type: Review.
[66] Hidalgo, M. — Analytical role: PDX models as translational cancer-research platforms. Evidence type: Review.
[67] Neal, J.T. — Analytical role: Air–liquid-interface organoids retaining endogenous immune cells; checkpoint response. Evidence type: Patient-derived/mouse perturbation.
[68] Voabil, P. — Analytical role: Ex vivo PD-1 response in tumor fragments and concordance with clinical response. Evidence type: Patient-derived perturbation + clinical association.
[69] Bruna, A. — Analytical role: Breast-cancer xenografts and short-term cultures for compound screening. Evidence type: Patient-derived preclinical platform.
[70] Drost, J. — Analytical role: Organoid culture, cancer modeling and patient-derived drug testing. Evidence type: Review.
[71] Tiriac, H. — Analytical role: Pancreatic organoid drug profiles and chemotherapy-response signatures. Evidence type: Patient-derived perturbation + association.
[72] Kopper, O. — Analytical role: Ovarian organoids preserving heterogeneity and enabling drug testing. Evidence type: Patient-derived platform.
[73] Jung, J. — Analytical role: Single-donor CRC liver-metastasis PDX and chemotherapy testing in immunodeficient mice. Evidence type: Patient-derived preclinical model.
[74] Ooft, S.N. — Analytical role: CRC organoids predicted irinotecan response, but not fluorouracil–oxaliplatin outcomes. Evidence type: Prospective response association.
[75] Vlachogiannis, G. — Analytical role: Metastatic gastrointestinal organoid drug responses compared with patient outcomes. Evidence type: Patient-derived response association.
[76] Esposito, A. — Analytical role: CRC immune-organoid resistance markers, independent staining and gene-knockout rescue. Evidence type: Patient-derived perturbation + external specimens.
[77] Biswas, N. — Analytical role: AI analysis of cancer multi-omics data. Evidence type: Review.
[78] Heo, Y.J. — Analytical role: Multi-omics integration, networks and cancer-subtype classification. Evidence type: Review.
[79] Raufaste-Cazavieille, V. — Analytical role: Multi-omics, precision oncology and immuno-oncology biomarkers. Evidence type: Review.
[80] Liu, M. — Analytical role: Technologies and translational prospects of three-dimensional tumor atlases. Evidence type: Review.
[81] Wu, S.Z. — Analytical role: Single-cell and spatial breast-cancer atlas and stromal–immune niches. Evidence type: Human observational/resource.
[82] Jackson, H.W. — Analytical role: Imaging mass cytometry and outcome-associated breast-cancer tissue architecture. Evidence type: Human observational.
[83] Helmink, B.A. — Analytical role: B-cell signatures, tertiary lymphoid structures and immunotherapy response. Evidence type: Human observational.
[84] Cabrita, R. — Analytical role: Tertiary lymphoid structures, T-cell states and melanoma outcomes. Evidence type: Human observational.
[85] Williams, H.L. — Analytical role: Spatial biomarkers for checkpoint-inhibitor response prediction. Evidence type: Review.
[86] Lan, Z. — Analytical role: Spatial-omics modalities, TME interactions and immunotherapy opportunities. Evidence type: Perspective/review.
[87] Addala, V. — Analytical role: Computational immunogenomic analysis of bulk and single-cell sequencing. Evidence type: Review.
[88] Greenwald, N.F. — Analytical role: Spatial/temporal biomarkers in the phase II TONIC cohort; on-treatment response prediction. Evidence type: Human longitudinal observational.
[89] Chen, J.H. — Analytical role: Lung-cancer stem-immunity hubs associated with PD-1-blockade outcomes. Evidence type: Human spatial observational.
[90] Argelaguet, R. — Analytical role: MOFA integration of shared and modality-specific multi-omics variation. Evidence type: Computational method.

[91] Cui, H. — Analytical role: scGPT cell representation, integration and perturbation-prediction tasks. Evidence type: Computational method.

[92] Hao, M. — Analytical role: scFoundation single-cell representation and downstream prediction benchmarks. Evidence type: Computational method.

[93] Aliazis, K. — Analytical role: Immune and non-immune TME compartments in checkpoint response and resistance. Evidence type: Review.

[94] Lambrechts, D. — Analytical role: Lung-cancer stromal-cell subtypes, independent profiling and survival associations. Evidence type: Human observational/resource.

[95] Kieffer, Y. — Analytical role: CAF subsets, Treg feedback and immunotherapy-resistance associations. Evidence type: Human profiling + functional assays.

[96] Jerby-Arnon, L. — Analytical role: Melanoma exclusion program, CDK4/6 inhibition and immunotherapy combinations. Evidence type: Human association + mouse perturbation.

[97] Sade-Feldman, M. — Analytical role: Melanoma T-cell states, TCF7 response prediction and exhaustion-targeting experiments. Evidence type: Human association + functional evidence.

[98] Xie, X. — Analytical role: Alternative modalities for conventionally difficult protein targets. Evidence type: Review.

[99] Hincapié-López, M. — Analytical role: Antisense versus antigene/TFO design requirements and validation gaps. Evidence type: Dual-method review.

[100] Wagner, J. — Analytical role: In situ bacterial chaperonin states measured by cryo-electron tomography. Evidence type: Structural experiments.

[101] Trott, O. — Analytical role: AutoDock Vina docking search and scoring. Evidence type: Method/tool.

[102] Friesner, R.A. — Analytical role: Glide conformational search and docking accuracy. Evidence type: Method/benchmark.

[103] Halgren, T.A. — Analytical role: Glide database-screening enrichment and scoring refinements. Evidence type: Benchmark.

[104] Irwin, J.J. — Analytical role: ZINC purchasable-compound libraries prepared for virtual screening. Evidence type: Compound resource.

[105] Morris, G.M. — Analytical role: AutoDock4 with selective receptor flexibility. Evidence type: Method/tool.

[106] Stärk, H. — Analytical role: EquiBind geometric pose prediction for supplied ligands. Evidence type: Computational method.

[107] Lu, W. — Analytical role: TANKBind interaction geometry, binding-site and affinity prediction. Evidence type: Computational method.

[108] Yang, K. — Analytical role: Learned molecular representations evaluated on public and industrial datasets. Evidence type: Benchmark/method.

[109] Mayr, A. — Analytical role: ChEMBL machine-learning comparison using nested cluster cross-validation. Evidence type: Benchmark.

[110] Schneuing, A. — Analytical role: DiffSBDD pocket-conditioned ligand generation and constrained redesign. Evidence type: Computational generation.

[111] Hu, Q. — Analytical role: DiffGui atom/bond diffusion, property guidance and selected wet-lab validation. Evidence type: Generation + experimental testing.

[112] Watson, J.L. — Analytical role: RFdiffusion protein-binder design; PD-L1 binding demonstrated, checkpoint efficacy unestablished. Evidence type: Protein design + binding assays.

[113] Brown, N. — Analytical role: GuacaMol distribution-learning and goal-directed molecular-generation evaluation. Evidence type: Benchmark.

[114] Polykovskiy, D. — Analytical role: MOSES molecular-generation validity, diversity and distribution metrics. Evidence type: Benchmark/resource.

[115] Olivecrona, M. — Analytical role: Reinforcement-learning optimization of sequence-based molecular generation. Evidence type: Computational generation.

[116] Walters, W.P. — Analytical role: Critical assessment of generative-AI claims in medicinal chemistry. Evidence type: Correspondence/perspective.

[117] Congreve, M. — Analytical role: 1,2,4-Triazine A2A antagonists, co-crystal structures and preclinical pharmacology; Parkinson's context. Evidence type: Structure-based discovery.

[118] Borodovsky, A. — Analytical role: AZD4635 A2A structure, binding, signaling, immune function and mouse tumor response. Evidence type: Structure + preclinical pharmacology.

[119] Cho, B.C. — Analytical role: Bintrafusp alfa versus pembrolizumab; no demonstrated superiority and more severe adverse events. Evidence type: Randomized phase III.

[120] Chen, B. — Analytical role: CADD methods applied to Chinese materia medica research. Evidence type: Review.

[121] Dewhirst, M.W. — Analytical role: Tumor drug transport, perfusion, diffusion and binding/uptake constraints. Evidence type: Review.

[122] Cournia, Z. — Analytical role: Relative binding free-energy implementation and practical validity conditions. Evidence type: Methods review/application synthesis.

[123] Aldeghi, M. — Analytical role: Absolute binding free energies for BRD4 inhibitors, including pseudo-prospective evaluation. Evidence type: Computational benchmark.

[124] Ross, G.A. — Analytical role: Grand canonical Monte Carlo improved buried-water sampling in free-energy calculations. Evidence type: Sampling method/benchmark.

[125] Brunk, E. — Analytical role: QM/MM molecular dynamics in ground and electronically excited states. Evidence type: Review.

[126] Pires, D.E.V. — Analytical role: pkCSM pharmacokinetic and toxicity prediction from molecular signatures. Evidence type: Prediction tool.

[127] Xiong, G. — Analytical role: ADMETlab 2.0 multi-endpoint ADMET prediction. Evidence type: Prediction tool.

[128] Lipinski, C.A. — Analytical role: Solubility, permeability and physicochemical rules in drug development. Evidence type: Review/methodology.

[129] Daina, A. — Analytical role: SwissADME physicochemical, pharmacokinetic and medicinal-chemistry estimates. Evidence type: Prediction tool.

[130] Fu, L. — Analytical role: ADMETlab 3.0 broader endpoints, uncertainty and programmatic access. Evidence type: Prediction tool.

[131] Banerjee, P. — Analytical role: ProTox-II toxicity-endpoint prediction and validation. Evidence type: Prediction tool.

[132] Cumming, J.G. — Analytical role: QSAR-guided potency, selectivity and ADMET optimization. Evidence type: Review.

[133] Waring, M.J. — Analytical role: Cross-company attrition and associations with compound properties and safety. Evidence type: Industrial retrospective analysis.

[134] Cook, D. — Analytical role: AstraZeneca project analysis and the five-R development framework. Evidence type: Industrial retrospective analysis.

[135] Morgan, P. — Analytical role: Exposure, target binding and functional pharmacology in phase II survival. Evidence type: Industrial retrospective analysis.

[136] Wilhelm, S. — Analytical role: Nanoparticle tumor-delivery efficiency and biological transport barriers. Evidence type: Literature analysis/perspective.

[137] Rosenblum, D. — Analytical role: Targeted delivery approaches and clinical-translation constraints. Evidence type: Review.

[138] Sindhwani, S. — Analytical role: Transendothelial nanoparticle entry into tumors across tested models. Evidence type: Transport experiments.

[139] Mitchell, M.J. — Analytical role: Precision nanoparticle design for heterogeneous delivery barriers. Evidence type: Review.

[140] Liu, Z. — Analytical role: FAP-responsive FNIII14 nanocarrier, CAF matrix modulation and tumor penetration. Evidence type: Preclinical delivery.

[141] Lu, X. — Analytical role: Peptide PROTAC nanoparticles targeting STAT3/β-catenin and CRC immune responses. Evidence type: Preclinical degradation.

[142] Petzold, G. — Analytical role: Lenalidomide-dependent CRBN recruitment of CK1α for degradation. Evidence type: Structural mechanism.

[143] Liu, F. — Analytical role: AI integration of multi-omics and clinical cancer data. Evidence type: Review.

[144] Kline, A. — Analytical role: Multimodal machine learning in precision health and deployment gaps. Evidence type: Scoping review.

[145] Stahlschmidt, S.R. — Analytical role: Deep-learning fusion strategies for biomedical data. Evidence type: Review.

[146] Jiménez-Luna, J. — Analytical role: Explainable AI methods, applications and interpretability limits in drug discovery. Evidence type: Review.

[147] Vamathevan, J. — Analytical role: Machine-learning applications and validation challenges in drug discovery. Evidence type: Review.

[148] Truhn, D. — Analytical role: Large language models and multimodal foundation models in precision oncology. Evidence type: Editorial.

[149] Niu, C. — Analytical role: Foundation models, multimodality, reasoning and human oversight in cancer management. Evidence type: Comment.

[150] Hao, Y. — Analytical role: LLM use and evaluation in oncology decision support. Evidence type: Systematic review/meta-analysis.

[151] Sutanto, H. — Analytical role: AI-assisted small-molecule cancer immunomodulation. Evidence type: Perspective.

[152] Schneider, G. — Analytical role: Grand challenges for AI-assisted small-molecule discovery. Evidence type: Expert perspective.

[153] Hernán, M.A. — Analytical role: Causal estimands, exchangeability, positivity, consistency and longitudinal inference. Evidence type: Methods textbook.

[154] Pearl, J. — Analytical role: Transportability of causal effects across populations using selection diagrams. Evidence type: Causal methodology.

[155] Murphy, S.A. — Analytical role: Optimal dynamic treatment rules from experimental or observational data. Evidence type: Statistical methodology.

[156] Laber, E.B. — Analytical role: Dynamic-regime inference, nonregularity and adaptive confidence intervals. Evidence type: Statistical methodology.

[157] Williams, N.T. — Analytical role: Learning longitudinal treatment rules; opioid-treatment illustration, not oncology validation. Evidence type: Methods tutorial/application.

[158] Zhao, Y. — Analytical role: Anticancer drug–drug interaction prediction; safety/interaction evidence, not synergy validation. Evidence type: Systematic review.

[159] Yadav, B. — Analytical role: ZIP model and dose–response interaction landscapes. Evidence type: Synergy method.

[160] Ianevski, A. — Analytical role: SynergyFinder 2.0 analysis and visualization of combination-response surfaces. Evidence type: Analysis tool.

[161] Bliss, C.I. — Analytical role: Independent joint action and departures from expected combined toxicity. Evidence type: Foundational methodology.

[162] Berenbaum, M.C. — Analytical role: Definitions and quantitative interpretation of drug synergy. Evidence type: Methods review.

[163] Menden, M.P. — Analytical role: Cancer drug-combination prediction across cell lines and combinations. Evidence type: Community benchmark.

[164] Li, L. — Analytical role: Deep-learning methods for anticancer drug-synergy prediction. Evidence type: Review.

[165] Holbeck, S.L. — Analytical role: NCI-ALMANAC drug pairs, cell-line screening and selected xenograft follow-up. Evidence type: Screening resource + preclinical follow-up.

[166] Peng, L. — Analytical role: CAR-T/CAR-NK therapy and barriers in solid tumors. Evidence type: Review.

[167] Galon, J. — Analytical role: Immune hot, altered and cold tumors and combination-immunotherapy strategies. Evidence type: Review.

[168] Shalhout, S.Z. — Analytical role: Oncolytic-virus pharmacology, clinical experience and combination challenges. Evidence type: Review.

This reference map records how methodological, biological, patient-derived, pharmacological, and clinical sources supported different analytical purposes. Methodological relevance was not treated as evidence of therapeutic efficacy, observational association was not equated with an intervention effect, and patient-derived perturbation was not assumed to establish model-to-human transportability. The map preserves these evidence boundaries and makes the use of each source explicit.
